# A Case Study from the Overexpression of *OsTZF5*, Encoding a CCCH Tandem Zinc Finger Protein, in Rice Plants Across Nineteen Yield Trials

**DOI:** 10.1186/s12284-024-00705-z

**Published:** 2024-04-09

**Authors:** Alexandre Grondin, Mignon A. Natividad, Takuya Ogata, Asad Jan, Amélie C. M. Gaudin, Kurniawan R. Trijatmiko, Evelyn Liwanag, Kyonoshin Maruyama, Yasunari Fujita, Kazuko Yamaguchi-Shinozaki, Kazuo Nakashima, Inez H. Slamet-Loedin, Amelia Henry

**Affiliations:** 1 Rice Breeding Innovations Department, International Rice Research Institute, Pili Drive, Los Baños, Laguna Philippines; 2 Biological Resources and Post-Harvest Division, Japan International Research Center for Agricultural Sciences (JIRCAS), Tsukuba, Ibaraki 305-8686 Japan; 3 Food Program, Japan International Research Center for Agricultural Sciences (JIRCAS), Tsukuba, Ibaraki 305-8686 Japan; 4 Present Address: Institut de Recherche Pour Le Développement, Université de Montpellier, UMR DIADE, 911 Avenue Agropolis, 34394 Montpellier, France; 5 Present Address: Institute of Biotechnology and Genetics Engineering, The University of Agriculture, Peshawar, 25130 Khyber Pakhtunkhwa Pakistan; 6 Present Address: Department of Plant Sciences, University of California Davis, Davis, CA 95616 USA; 7 Present Address: Laboratory of Plant Molecular Physiology, The University of Tokyo, Tokyo, 113-8657 Japan; 8 Tokyo University of Agriculture, Research Institute for Agricultural and Life Sciences, Tokyo, Japan

**Keywords:** Rice, Transgenic, Drought, Yield, Transgene expression

## Abstract

**Background:**

Development of transgenic rice overexpressing transcription factors involved in drought response has been previously reported to confer drought tolerance and therefore represents a means of crop improvement. We transformed lowland rice IR64 with *OsTZF5*, encoding a CCCH-tandem zinc finger protein, under the control of the rice *LIP9* stress-inducible promoter and compared the drought response of transgenic lines and nulls to IR64 in successive screenhouse paddy and field trials up to the T_6_ generation.

**Results:**

Compared to the well-watered conditions, the level of drought stress across experiments varied from a minimum of − 25 to − 75 kPa at a soil depth of 30 cm which reduced biomass by 30–55% and grain yield by 1–92%, presenting a range of drought severities. *OsTZF5* transgenic lines showed high yield advantage under drought over IR64 in early generations, which was related to shorter time to flowering, lower shoot biomass and higher harvest index. However, the increases in values for yield and related traits in the transgenics became smaller over successive generations despite continued detection of drought-induced transgene expression as conferred by the *LIP9* promoter. The decreased advantage of the transgenics over generations tended to coincide with increased levels of homozygosity. Background cleaning of the transgenic lines as well as introgression of the transgene into an IR64 line containing major-effect drought yield QTLs, which were evaluated starting at the BC_3_F_1_ and BC_2_F_3_ generation, respectively, did not result in consistently increased yield under drought as compared to the respective checks.

**Conclusions:**

Although we cannot conclusively explain the genetic factors behind the loss of yield advantage of the transgenics under drought across generations, our results help in distinguishing among potential drought tolerance mechanisms related to effectiveness of the transgenics, since early flowering and harvest index most closely reflected the levels of yield advantage in the transgenics across generations while reduced biomass did not.

**Supplementary Information:**

The online version contains supplementary material available at 10.1186/s12284-024-00705-z.

## Background

Transgenic versions of rice, potato, wheat, and peanut with enhanced drought tolerance have been reported in the literature during the last decade (Dubouzet et al. 2003; Pellegrineschi et al. 2004; Oh et al. 2005; Behnam et al. 2006; Oh et al. 2009; Bhatnagar-Mathur et al. 2013). Concurrently, molecular analyses have shown that drought triggers signaling pathways involving many genes, and physiological analyses suggest that drought tolerance likely involves multiple mechanisms acting in combination (Nakashima et al. 2014; Henry et al. 2015). Efforts to identify promoters with specific activation under drought stress that may be suitable for maintaining yield under both drought and irrigated conditions have also been pursued (Maruyama et al. 2012). Therefore, the challenge in transgenic rice studies for improving drought resistance is to translate molecular knowledge to appropriately express genes that can lead to improvement of crops grown in fields.

A few examples of rice plants overexpressing a galactinol synthase or different NAC-type transcription factors showed improved yield or yield related-traits as compared to the wild types when grown in field conditions under drought stress (Jeong et al. 2010, 2013; Redillas et al. 2012; Lee et al. 2016; Shim et al. 2018; Selvaraj et al. 2017; for recent review see Khan et al. 2020). Other transcription factors such as zinc finger proteins have been suggested to improve tolerance to abiotic stresses (Maruyama et al. 2012; Jan et al. 2013). Overexpression of the petunia gene *ZPT2-3* encoding a Cys_2_/His_2_-type zinc finger protein conferred enhanced dehydration tolerance in transgenic petunia (Sugano et al. 2003). Furthermore, overexpression of the *OsSAP1* gene encoding a rice zinc finger protein in tobacco conferred better germination and seedling growth under cold, dehydration, and salt stress (Mukhopadhyay et al. 2004). The overexpression of *OsTZF1* in rice (cv Nipponbare), a gene encoding a CCCH-tandem zinc finger protein, also conferred tolerance to salt and drought in transgenic plants grown in pots (Jan et al. 2013).

In rice, 67 genes encoding CCCH-tandem zinc finger proteins were identified (Wang et al. 2008) and many among them are induced by drought stress (Maruyama et al. 2012). *OsTZF1*, which is widely expressed in rice, localizes in the cytoplasm under stress, where it likely regulates stress-related genes through the control of RNA metabolism (Zhang et al. 2012; Jan et al. 2013). Similarly, overexpression of *OsTZF5*, a close homolog to *OsTZF1*, in upland rice varieties Curinga and Nerica4 under the drought-inducible promoter *OsNAC6*, improved plant performance under drought at multiple growth stages (Selvaraj et al. 2020). It is likely that, like *OsTZF1*, *OsTZF5* triggers the expression of genes that improve drought tolerance. However, the physiological mechanisms involved in the improved drought tolerance conferred by both genes remain unknown.

In this study, we transformed the lowland rice variety IR64 with the *OsTZF5* gene (OsC3H33; Os05g0128200/LOC_Os05g03760) under the control of the rice *LIP9* drought-inducible promoter (Maruyama et al. 2012). Our aim was to characterize the agronomic and physiological effects of *OsTZF5* transgene expression under drought in rice. In this regard, fertile IR64 transgenic lines with single or low T-DNA copies carrying the *OsTZF5* transgene were selected for: (1) molecular characterization of the transgene expression and insertion site and (2) yield and physiological characterization under drought and well-watered conditions in comparison with transformed non-transgenic lines (nulls) and background-cleaned lines. We hypothesized that any transgene improvement of yield and related traits would be heritable and could provide insight into breeding strategies to improve drought tolerance in rice. However, over the course of our experiments we observed generational effects on the performance of the *OsTZF5* transgenics and we therefore focused on identifying the physiological traits that corresponded most to the trends in yield advantage.

## Material and Methods

### Plant Material

Three sets of lines were used in this study: transgenic lines (the original set of lines transformed with *LIP9:OsTZF5*), background-cleaned lines (from three transgenic events that were backcrossed to the wild type variety), and pyramided lines (from crossing of the lead transgenic event with a breeding line in the same background).

*Agrobacterium tumefaciens* (LBA4404) containing the pBIH plasmid vector carrying the hygromycin phosphotransferase (HPT) gene and the ORF of *OsTZF5 (Os05g0128200)* controlled by rice *LIP9* promoter (*LIP9:OsTZF5*) were used to transform immature IR64 embryos by co-cultivation (see Additional file 1 for sequence information). Thirty-nine independent T_0_ lines with single or low copy numbers were regenerated and transferred to the greenhouse, and nine lines showing no morphological defects were selected for further screening as described by Hiei and Komari (2006). Transgenic lines were selected based on their resistance to hygromycin and presence of the transgene after PCR amplification (Additional file 2: Table S1; Fig. S1A). The zygosity of T_1_ plants was determined by PCR on T_2_ progeny using the selectable marker (*HPT*) gene. The percentage of plants that were homozygous for the transgene became closer to 100% with each successive generation (Additional file 2: Fig. S1B). Based on the yield of segregating T_1_ and T_2_ transgenic lines under well-watered and drought conditions, three lines were selected for further analysis in screenhouse, greenhouse cylinder, and field trials: 1-TZF5-13, 1-TZF5-24 and 1-TZF5-72 (Table 1). Azygous nulls of these transgenic lines (harvested from SH4-W) were subjected to PCR and Southern blot analysis, from which no T-DNA could be detected (Additional file 2: Fig. S2). Three azygous null segregants were selected (0-TZF5-13, 0-TZF5-24 and 0-TZF5-72) and included in some screenhouse, field, and cylinder trials depending on seed availability (Table 1). Since the nulls were limited across experiments, the results are presented separately in the Supplemental files. In all trials, IR64 (the wild type) and IR77298-14-1-2-10 (14-1-2-10 BIL), a drought-tolerant line from the IRRI marker-assisted drought breeding program with QTLs *qDTY*_*2.2*_ and *qDTY*_*4.1*_ in the IR64 background (Swamy et al. 2013), were used as check varieties.

**Table 1:** Description of trials

Environment season	Lines included	Generation	Trial	Treatment	Draining date (das)	Temp (ave.°C)
*Transgenic trials*						
Screenhouse DS2011	IR64, 1-TZF5-13, 1-TZF5-24, 1-TZF5-72	T_1_	**SH1-W**	Well-watered	–	28
			**SH1-VRS**	Veg + Repro Stress	44	29
Field DS2012	IR64, 0-TZF5-24, 1-TZF5-13, 1-TZF5-24, 1-TZF5-72, 14-1-2-10 BIL	T_2_	**F-W**	Well-watered	–	28
			**F-VRS**	Veg + Repro Stress	45	28
			**F-RS**	Repro Stress	60	28
Screenhouse WS2012	IR64, 1-TZF5-72, 14-1-2-10 BIL	T_3_	**SH2-W**	Well-watered	–	27
			**SH2-VRS**	Veg + Repro Stress	36	28
Screenhouse WS2013	IR64, 0-TZF5-13, 0-TZF5-24, 0-TZF5-72, 1-TZF5-13, 1-TZF5-24, 1-TZF5-72, 14-1-2-10 BIL	T_2_–T_5_	**SH3-W**	Well-watered	–	
			**SH3-VRS**	Veg + Repro Stress	53	
Screenhouse DS2015	IR64, 0-TZF5-13, 0-TZF5-24, 0-TZF5-72, 1-TZF5-13, 1-TZF5-24, 1-TZF5-72, 14-1-2-10 BIL	T_3_–T_5_	**SH4-W**	Well-watered	–	27
			**SH4-VRS**	Veg + Repro Stress	49	28
Screenhouse DS2014	IR64, 1-TZF5-13, 1-TZF5-24, 1-TZF5-72, 14-1-2-10 BIL	T_4_–T_6_	**SH5-W**	Dry direct seeded, Well-watered	–	
			**SH5-SS**	Dry direct seeded, Seedling Stress	10	
Cylinder WS2013	IR64,0-TZF5-13, 1-TZF5-13	T_3_–T_6_	**C1-W**	Well-watered	–	29
			**C1-VS**	Veg Stress	22	
Cylinder DS2014	IR64, 0-TZF5-24,0-TZF5-72, 1-TZF5-24,1-TZF5-72	T_3_–T_5_	**C2-W**	Well-watered	–	31
			**C2-VS**	Veg Stress	22	
*Background-cleaned trials*						
Cylinder WS2016	IR64, 0-TZF5-13, 0-TZF5-24,0-TZF5-72, 1-TZF5-13, 1-TZF5-24,1-TZF5-72, 3 BC_3_F_1_ lines (+), 3 BC_3_F_1_ lines (−),14-1-2-10 BIL	BC_3_F_1_	**C3-W**	Well-watered	–	28
			**C3-VS**	Veg Stress	26	28
Screenhouse WS2017	IR64, 0-TZF5-13, 0-TZF5-24,0-TZF5-72, 1-TZF5-13, 1-TZF5-24,1-TZF5-72, 3 BC_3_F_2_ lines (+), 3 BC_3_F_2_ lines (−),14-1-2-10 BIL	BC_3_F_2_	**SH6-W**	Well-watered	–	
			**SH6-RS**	Repro Stress	42	
*Pyramiding trials*						
Screenhouse WS2016	IR64, 1-TZF5-24,1-TZF5-72, 36 BC2F3 lines (+), 10 BC_2_F_3_ lines (−), IR87707-445-B-B-B	BC_2_F_3_	**SH7-W**	Well-watered	–	27
			**SH7-RS**	Repro Stress	48	28
Screenhouse DS2017	IR64, 1-TZF5-72, 9 BC_2_F_4_ lines (+), 1 BC_2_F_4_ lines (−), IR87707-445-B-B-B	BC_2_F_4_	**SH8-W**	Well-watered	–	31
			**SH8-RS**	Repro Stress	41	31
Screenhouse DS2018	IR64, 1-TZF5-72, 9 BC_2_F_5_ lines (+), 1 BC_2_F_5_ lines (−), IR87707-445-B-B-B	BC_2_F_5_	**SH9-W**	Well-watered	–	28
			**SH9-RS**	Repro Stress	56	28

*SH* Screenhouse,* F* Field,* C* Cylinder,* DS* dry season,* WS* wet season

IR64: drought susceptible wild-type, 14-1-2-10 BIL and IR87707-445-B-B-B: drought tolerant QTL lines in the background of IR64, 1-TZF5-13, 1-TZF5-24, 1-T-TZF5-72: transgenic lines, 0-TZF5-13, 0-TZF5-24, 0-T-TZF5-72: azygous null lines, W: well-watered, RS: reproductive stage drought stress, VRS: vegetative and reproductive stage drought stress, VS: vegetative stage drought stress, SS: seedling stage drought stress, das: days after sowing

Background cleaning of the transgenic lines was conducted by backcrossing 1-TZF5-13 (T5), 1-TZF5-24 (T5) and 1-TZF5-72 (T3) to IR64 three times (Additional file 2: Fig. S3). Crossing was performed according to the procedure described by Jennings et al. (1979) during the 2014 dry season (DS) in a contained screenhouse. IR64 was used as the female parent, and emasculated spikelets were pollinated with the male parents 1-TZF5-13, 1-TZF5-24, or 1-TZF5-72 before covering with a glassine bag and allowing the seeds to mature for 20–25 days. Presence of the transgene was ensured by PCR genotyping using event-specific primers (Additional file 2: Tables S2 and S3).

Pyramiding of the 1-TZF5-72 transgenic line with major-effect drought yield QTL was performed by crossing, during the 2014 wet season (WS). IR87707-445-B-B-B was used as the female parent and 1-TZF5-72 (T5) was used as the male parent as described above (Additional file 2: Fig. S4). IR87707-445-B-B-B is derived from a cross between IR64 and Aday Sel which possesses major-effect drought-yield QTLs *qDTY*_*2.2*_ and *qDTY*_*4.1*_ (Swamy et al. 2013). Genotyping of the F_1_ progeny by PCR using the specific primers for *qDTY*_*2.2*_ and *qDTY*_*4.1*_ and *LIP9:TZF5* (Additional file 2: Table S3) was conducted to ensure that all three desired alleles were present (Additional file 2: Table S4). Pyramiding lines were subsequently backcrossed to IR64 and a subset of 10 BC_2_F_2_ lines with all three introgressions were selected and advanced to BC_2_F_5_ by selfing (Additional file 2: Fig. S4). Polymorphisms between 1-TZF5-72, IR87707-445-B-B-B, the BC_2_F_5_ pyramided lines, the background cleaned lines and IR64 were analyzed after Infinium 6 k or 7 k SNP genotyping conducted at the IRRI Genotyping Services Laboratory.

### Flanking Sequence Analysis of the T-DNA Insertion

The flanking sequences of the T-DNA insertions in lines 1-TZF5-13, 1-TZF5-24, and 1-TZF5-72 were determined by the thermal asymmetric interlaced (TAIL) PCR as described by Liu et al. (1995). Genomic DNA was isolated using ISOPLANT II (Nippon Gene, Toyama, Japan) according to the manufacturer’s instructions. TAIL-PCR products were sequenced and locations of the T-DNA insertion were determined for each transgenic line using the BLAST server in Rice Genome Annotation Project (http://rice.uga.edu/), which were on chromosome 10 in 1-TZF5-13, chromosome 7 in 1-TZF5-24, and chromosome 3 in 1-TZF5-72 (Additional file 1: Fig. S5). TAIL-PCR results were verified using primers designed for each allele (Additional file 2: Table S5).

Southern blot analyses were performed using 5 μg of genomic DNA extracted according to the CTAB method (Murray and Thompson 1980) and digested with *Eco*RI, *Bam*HI, or *Eco*RI plus *Bam*HI restriction enzymes at 37 °C overnight. Digested genomic DNA was resolved by electrophoresis on a 0.7% agarose gel in Tris–acetate-EDTA buffer and transferred to a Biodyne B nylon membrane (Nihon Pall, Tokyo, Japan) as described by Southern (1975). The membrane was hybridized with a ^32^P-labeled DNA probe and the autoradiograph was scanned on a Typhoon FLA 7000 (GE Healthcare, Uppsala, Sweden). The 1.5 kb *Pvu*II/*Xho*I fragment from the plasmid pBIH-LIP9:OsTZF5 was labeled with [α-^32^P]-dCTP with the BcaBEST labeling kit (Takara Bio, Shiga, Japan) and used as a *HPT* gene probe.

### Screenhouse and Field Trials

Screenhouse (SH) trials and field (F) trials were performed at the International Rice Research Institute (Los Baños, Philippines; 14° 10′ 11.81″ N, 121° 15′ 39.22″ E) according to Gaudin et al. (2013) (Table 1). These trials included a well-watered treatment, which was maintained flooded until plant maturity (SH1-W, SH2-W, SH3-W, SH4-W and F-W), and a drought stress treatment initiated at vegetative stage (SH1-VRS, SH2-VRS, SH3-VRS, SH4-VRS and F-VRS) or at reproductive stage (F-RS) until maturity. One dry direct-seeded trial was also included to test the response of the transgenics to drought at seedling stage (SH5-SS) in comparison with the corresponding irrigated trial (SH5-W). In general, each successive transgenic trial represented an advance of one generation for each event, however due to seed availability, some seed sources from the same generation were grown in consecutive trials. Background-cleaned lines at the BC_3_F_2_ generation were evaluated in screenhouse trial 6 (SH6-W and SH6-RS) (Table 1). Pyramided lines were grown in screenhouse trials 7, 8 and 9 (SH7-W, SH7-RS, SH8-W, SH8-RS, SH9-W, and SH9-RS). In Trial SH7, 49 BC_2_F_3_ lines were planted from which 10 lines were selected based on the highest grain yield for each QTL/transgene combination available and included in Trials SH8 (BC_2_F_4_) and SH9 (BC_2_F_5_).

The SH1 screenhouse trial was arranged in an alpha lattice design while all other trials were arranged in randomized complete block designs, with 1.2-m^2^ plots for Trial SH7, 0.6–1.2-m^2^ plots (2–3 rows of 5–8 plants) for Trials SH1, SH5, and SH6, and 1.8–2.25 m^2^ plots (3 rows of 12–15 plants) for Trials SH2, SH3, SH4, SH6, SH8, and SH9. Each trial contained three to four replications per genotype. The screenhouse was composed of two independent 1-m deep intact flooded soil beds (8 m wide × 25 m long) separated by a 1.5 m-wide concrete alley and lined with a black plastic sheet to avoid leakage. The soil bed in which the drought treatment was planted contained perforated pipes at a 1 m depth that were connected to two spillways located at each side of the screenhouse, and was protected from rainfall by a transparent roof. The soil in both treatments was maintained flooded until the drought stress treatment was initiated (at around 40 days after sowing, DAS) by withholding irrigation and soil draining by pumping water from the spillways at both sides of the soil bed. The drought stress treatment was not re-watered during the screenhouse trials, except in Trial SH1-VRS (re-watered at 102 DAS) and SH8-RS (re-watered at 80 DAS).

The field trial was arranged in an alpha lattice design with four replications of 3-m^2^ plots (4 rows of 15 hills). As a biosafety measure, five rows of *Sesbania herbacea* were planted around the experimental field area as a pollen trap. Drought stress treatments were initiated during the vegetative stage (45 DAS) or during the reproductive stage (60 DAS) by withholding irrigation. The drought stress treatments were interrupted by rainfall at 94 DAS and no further irrigation was applied. Soil moisture was monitored after initiating the drought stress in all screenhouse and field trials using tensiometers (Soilmoisture Equipment Co., USA) installed at a depth of 30 cm (Additional file 2: Fig. S6).

For all screenhouse and field trials, seeds were germinated in the dark on moistened filter paper in Petri dishes at 33 °C for 3 days, then sown in 104-well trays filled with fertilized soil (14N-14P-14K) at the rate of 30 kg N ha^−1^. Twenty-one-day-old single seedlings were pulled and transplanted into puddled paddy soil with 25-cm spacing between rows and 20 cm between hills. When the number of transgenic seedlings was insufficient, IR64 was transplanted to fill the plots but was excluded from the physiology or yield measurements. Basal fertilizer was applied before transplanting using complete fertilizer (14N-14P-14K) at the rate of 40 kg N ha^−1^, and a topdressing of 50 kg N ha^−1^ ammonium sulfate was applied before panicle initiation. Manual weeding was done regularly in all trials. As needed, mollusk pests were controlled with niclosamide (0.25 L ha^−1^) and saponin (20 kg ha^−1^) (Biosolutions International Corp., Quezon City, Metro Manila, Philippines) and insect pest were controlled with Prevaton (0.76 L ha^−1^), Cartap (0.96 kg ha^−1^) and Provadon (1.92 L ha^−1^) (Quezon Farmers Agricultural Supply, Alaminos, Laguna, Philippines).

### Growth and Water Uptake Measurements in Cylinder Trials

Experiments in soil-filled cylinders (C) were performed on the transgenic lines (Trials C1 and C2) and background-cleaned lines (Trial C3) under well-watered and gradual dry-down conditions using nulls and IR64 as checks. Cylinders were arranged on tables within the screenhouse in a randomized complete block design with four replications. The cylinders (36 cm height and 20 cm diameter) were filled with 8.5 kg of soil fertilized at a rate of 0.3 g kg^−1^ (14N-14P-14 K). Two drainage holes at the bottom of each cylinder were plugged to keep the soil saturated during plant establishment. In the well-watered treatment, the soil was kept well-irrigated throughout the trials. In the drought stress treatment, dry-down was initiated at around 23–25 DAS by removing the plug from the drainage holes and the cylinders were covered around the stems with transparent plastic sheets to minimize soil evaporation. Target weights were calculated to allow a gradual and uniform drydown (so as not to exceed a rate of about 5% per day) until the cylinders reached 20% of field capacity over three weeks. Cylinders were weighed three times per week and water was added to reach the target weight if needed.

In both treatments, images of the shoots were taken at the time of weighing and leaf area was measured by color thresholding in *ImageJ* software V 1.45 according to Kijoji et al. (2012). Water uptake by each plant was calculated from the difference in cylinder weight between successive weighing dates, and was used to calculate cumulative water uptake from the start of the dry-down period until the harvest (at 56, 67, and 61 DAS in Trials C1, C2, and C3-VS, respectively). Water uptake rates were calculated as the amount of water uptake divided by the number of days between two successive weighing and normalized by leaf area.

### Plant Water Status Related Measurements

Relative water content (RWC), leaf water potential (LWP) and leaf osmotic potential (LOP) were measured on the youngest fully expanded leaves during the cylinder trial C2. RWC was measured as: (fresh leaf weight under stress-leaf dry weight)/(turgid leaf weight-leaf dry weight) × 100. The turgid weight was obtained after soaking the leaf overnight in water, after which the leaf was dried in an oven to obtain the dry weight. LWP was measured by inserting three leaves per plot into a pressure chamber (Soilmoisture Equipment Corp., USA) at mid-day and recording the minimum pressure at which outward sap flux was observed. Leaves used for LWP were further frozen in a 5-ml syringe at − 15 °C for LOP measurements. LOP was measured on 10 µl of sap (pressed from thawed leaf tissue) using a vapor pressure osmometer (Vapro model 5520, Wescor, Logan, UT, USA). Other plant water status-related measurements such as canopy temperature, stomatal conductance, photosynthesis, quantum yield of photochemical energy, abscisic acid content and root length density at depth were measured in multiple trials; these procedures are described in the legends of the supplemental figures.

### Stem Carbohydrate Content Measurements

Stem carbohydrate content was measured after drought stress initiation in Trials SH4 and SH6 on three stems (culm + leaf sheath) from randomly sampled plants in each plot. Stem samples were oven-dried at 70 °C for 3 days. At each sampling date, stem samples were pooled and 200 mg of finely-ground tissue was used for determination of ethanol-soluble sugar concentration according to the protocol described by Ismail et al. (2009). Briefly, stem soluble sugar were extracted in 80% ethanol and concentration was quantified by a colorimetric assay using anthrone reagent (Sigma-Aldrich, Missouri, USA; Fales 1951).

### Phenology and Grain Yield Measurements

Days to flowering and plant height were recorded when 50% of the rice plants in the plot reached flowering. Tiller number, panicle number, and shoot dry weight were measured at harvest on three plants per plot. Seeds harvested from these three plants were oven dried for three days at 42 °C and weight was normalized to a 14% moisture content to calculate grain yield. Harvest index was calculated as: grain weight/(grain weight + shoot dry weight). Harvest data could not be reported from SH6 due to rodent damage before sample processing.

### Transgene Expression Analysis by Northern Blot and Quantitative PCR

To characterize transgene expression, leaves were sampled from transgenic lines in Trial C2 (generation T_3_ for 1-TZF5-72 and T_5_ for 1-TZF5-13 and 1-TZF5-24) at 38 DAS (16 days after drought initiation), and RNA was isolated from leaf tissue using TRIZOL reagent (Invitrogen, USA) according to the manufacturer’s instructions. In addition, seeds of the transgenic lines and three pyramided lines were grown (generation T_4_ for 1-TZF5-72, T_6_ for 1-TZF5-13 and 1-TZF5-24, and BC_2_F_5_ for the pyramided lines) at the Japan International Research Center for Agricultural Sciences (JIRCAS, Tsukuba, Japan) to generate tissue for northern blot and/or quantitative PCR analyses. Seeds were sown directly into open-bottomed 50 ml plastic tubes filled with soil in a greenhouse. For the transgenic lines, plants were grown under well-irrigated conditions until 32 DAS, followed by a rapid drought stress that was induced by withholding water for 3 days. For the pyramided lines, plants were grown under well-irrigated conditions until 14 DAS, after which water was withheld for 4 days. Leaves from 3 plants were combined and total RNA was isolated from leaf tissue using RNAiso Plus reagent (Takara Bio, Japan) according to the manufacturer’s instructions at 32, 34 and 35 DAS in the transgenic lines and 14, 17, and 18 DAS in the pyramided lines.

For real-time quantitative PCR, RNA was subjected to a DNase treatment using a transcriptor first strand cDNA synthesis kit (Life Technologies, California, USA) or a RQ1 RNase-free DNase kit (Promega, USA) and cDNA was synthesized using a PrimeScript RT Master Mix (Takara Bio, Japan). Quantitative PCR was performed with a LightCycler® 480 system (Roche, Switzerland) using SYBR® Select Master Mix (Life Technologies, California, USA) at IRRI and with a 7500 real-time PCR system (Applied Biosystems, CA, USA) using SYBR Premix Ex Taq (Takara Bio, Japan) at JIRCAS. Primers are described in Additional file 2: Table S6.

For the northern blot analysis, RNA was transferred overnight from agarose gel to a Biodyne B nylon membrane (Nihon Pall, Japan) using 20× saline sodium citrate buffer. The membrane was hybridized with a ^32^P-labeled DNA probe and the autoradiograph was scanned on a Typhoon FLA 7000 (GE Healthcare, Sweden). The PCR fragment of the full length *OsTZF5* coding sequence was labeled with [α^32^P]-dCTP and used as a probe (Additional file 2: Table S7). 10 μg of total RNA was denatured by heating at 65 °C for 5 min in a sample buffer containing formaldehyde, formamide and ethidium bromide. The RNA samples were resolved by electrophoresis on an agarose gel containing formaldehyde in MOPS-borate-EDTA buffer, and the image for rRNA was taken with a UV transilluminator.

### Statistical Analysis

Statistical analyses were performed in R v. 2.15.1 (R Development Core Team 2017) and STAR v. 2.0.1 (http://bbi.irri.org/) using ANOVA to detect significant differences between lines and pairwise comparison using Least Significant Difference (LSD) test to class lines into significance groups. To detect genetic differences in leaf area and water uptake rates, a repeated measures analysis was conducted with the mixed model ASREML using Wald’s test in R, with genotype and date as fixed variables and replicate as a random variable.

## Results

### Selection of Drought Resistant Transgenic Lines

**Fig. 1:**
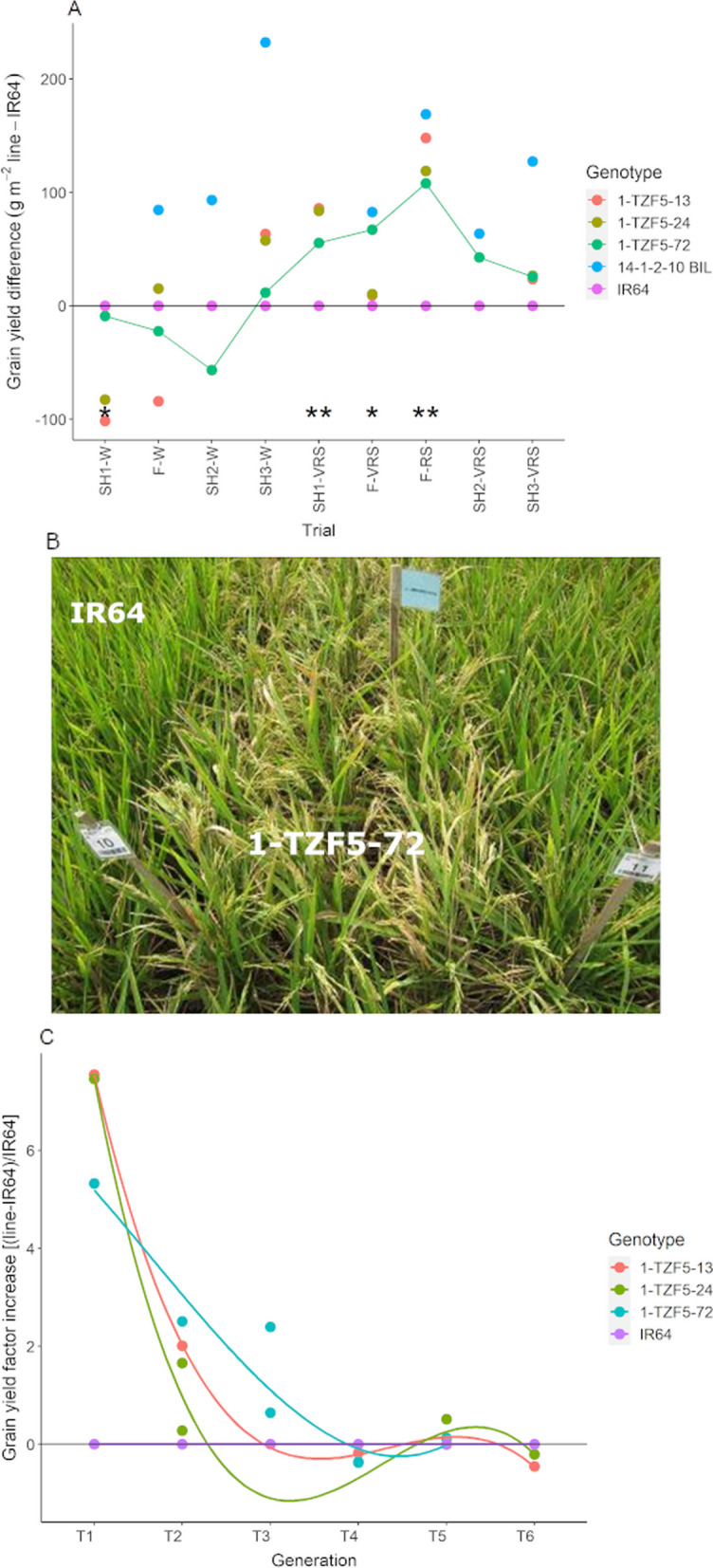
Transgenic lines—differences in grain yield compared to IR64 under **A** well-watered and drought stress conditions in initial transgenic screenhouse and field trials (see Table 1 for description of trials), **B** in trial F-VRS where line 1-TZF5-72 more panicles compared with IR64 at flowering time, **C** in transgenics across all drought screenhouse and field trials. Each point represents the difference in mean grain yield values (*n* = 4) between the corresponding line and IR64. Statistical analyses were performed on replicated grain yield values in **A**, and significant differences compared to IR64 are indicated by * (*p* < 0.05) and ** (*p* < 0.01). The mean values per generation in **C** overlapped across experiments and thus were not compared statistically.* SH* Screenhouse,* F* Field,* C* Cylinder,* DS* dry season,* WS* wet season. IR64: drought susceptible wild-type, 14-1-2-10 BIL: drought tolerant QTL line in the background of IR64, 1-TZF5-13, 1-TZF5-24, 1- T-TZF5-72: transgenic lines.* W* well-watered,* RS* reproductive stage drought stress,* VRS* vegetative and reproductive stage drought stress

In order to evaluate if *OsTZF5* transgenic lines (1-SCZF) showed improved drought tolerance, nine 1-TZF5 lines were evaluated under well-watered and drought stress conditions along with IR64 and 14-1-2-10 BIL. Three 1-TZF5 transgenic lines showed significantly higher grain yield than IR64 in Trials SH1-VRS and F-RS (*p* < 0.01), which was comparable to the grain yield of 14-1-2-10 BIL in Trial F-RS (Fig. 1A, B, Additional file 2: Fig. S7, and Table S8). The grain yield of the three 1-TZF5 transgenic lines under well-watered conditions was generally lower than in IR64, although these differences were not significant (Fig. 1A and Additional file 2: Table S8). Therefore, lines 1-TZF5-13, 1-TZF5-24 and 1-TZF5-72 were selected for further characterization.

### Environmental Conditions

Across yield trials, the environmental conditions and resulting levels of drought stress varied considerably in this study, with the most negative soil water potential values at a soil depth of 30 cm reaching below − 50 kPa in Trials SH2-VRS, F-VRS, and SH8-RS, while a more mild level of drought stress occurred in Trials F-RS, SH3-VRS, and SH7-RS with soil water potential values remaining above about − 30 kPa (Additional file 2: Fig. S6). Despite this variation, the level of stress can be considered relevant to agricultural conditions in drought-prone rice growing regions with some grain yield harvested in all yield trials. Biomass response to drought as compared to the biomass measured under irrigated treatment ranged from − 30% (SH5-SS) to + 55% (SH6-VS), indicating that in some studies the biomass was greater in the drought stress treatment. Yield reduction by drought ranged from 1% (SH7-RS) to 92% (F-VRS) (Additional file 2: Tables S8 and S9).

### Changes in Yield and Agro-morphological Traits Across Generations in the Transgenic Lines

To investigate the stability of the yield advantage of the transgenic lines over IR64 under drought (observed in SH1-3 and F; Fig. 1A), we measured yield-related traits along with agro-morphological traits in additional trials across generations of transgenic lines, while including the drought resistant check (14-1-2-10 BIL) and nulls as checks. Results of the three 1-TZF5 transgenic lines over a total of nine yield trials ranging up to the T_6_ generation were compiled. For the purpose of this study, “early generation” trials are those ranging from T_1_–T_3_, and “later generation” trials are those ranging from T_4_–T_6_. Across all trials, we observed a consistent decline in the yield advantage of the transgenic lines over IR64 under drought (Fig. 1C, Additional file 2: Tables S8 and S9) while the results in the well-watered treatment showed relatively stable performance of the transgenics in that they were typically slightly lower-yielding than IR64 (Additional file 2: Fig. S8). Yield of the azygous nulls generally remained above that of IR64 across generations (Additional file 2: Tables S8 and S9).

**Fig. 2:**
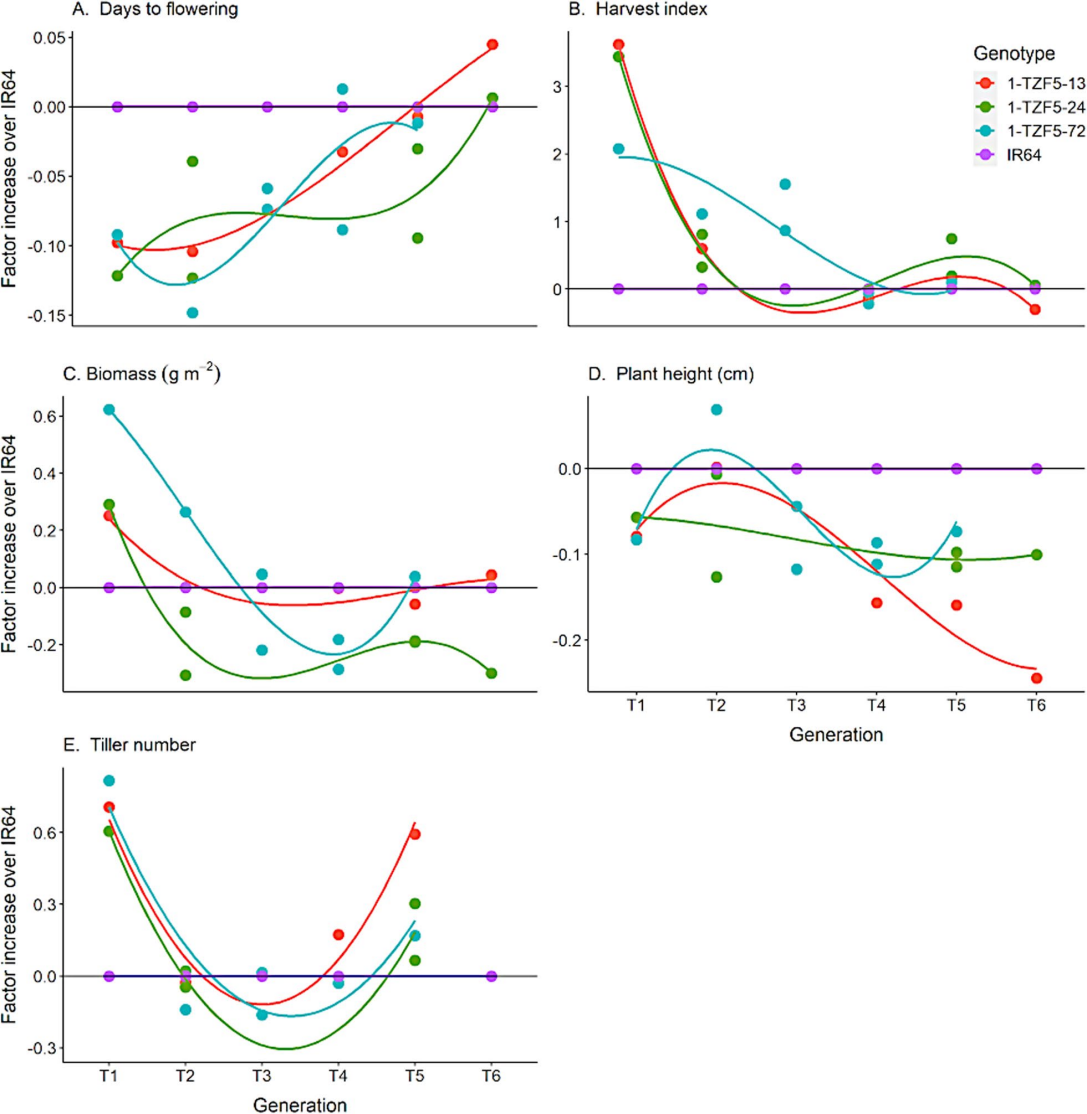
Transgenic lines—generational trends in agro-morphological traits across screenhouse and field trials under drought: **A** days to flowering, **B** harvest index, **C** straw biomass at harvest, **D** plant height, and **E** tiller number. Mean values per generation are shown which overlapped across experiments and thus were not compared statistically. IR64: drought susceptible wild-type, 1-TZF5-13, 1-TZF5-24, 1- T-TZF5-72: transgenic lines

Among agronomic traits, the days to flowering (DTF) and harvest index (HI) showed the most similar generational loss-of-effect trends to that of grain yield under drought (Fig. 2A, B, Additional file 2: Fig. S9), in which DTF of the transgenics was shorter in earlier generations, and HI was higher in earlier generations compared to IR64. Specifically, the three transgenic lines showed a significantly higher harvest index under drought than IR64 at the T_1_-T_2_ generations (Trials SH1-VRS and F-RS; *p* < 0.01 and *p* < 0.05 respectively, Additional file 2: Table S8), but the harvest index of the three transgenic lines was not significantly different than that of IR64 at the T_4_-T_6_ generations (Trials SH4-RS and SH5-SS; Additional file 2: Table S9). An increase in shoot biomass was observed under drought in the transgenic lines as compared to IR64 in the T_1_ generation, beyond which the biomass (and plant height) was generally smaller than that of IR64 (Fig. 2C, D). In contrast, tiller number was generally higher in the transgenics compared to IR64 across generations in both treatments (Fig. 2E, Additional file 2: Fig. S8). In well-watered treatments, the harvest index values in the transgenic lines did not vary significantly from the values observed in IR64 (Tables S8 and S9) while the biomass and plant height of the transgenics was consistently smaller than that of IR64 across generations. The transgenics did not show obvious generational trends in the well-watered treatments for any trait measured (Additional file 2: Fig. S8).

### OsTZF5 Expression in the Transgenic Lines

**Fig. 3:**
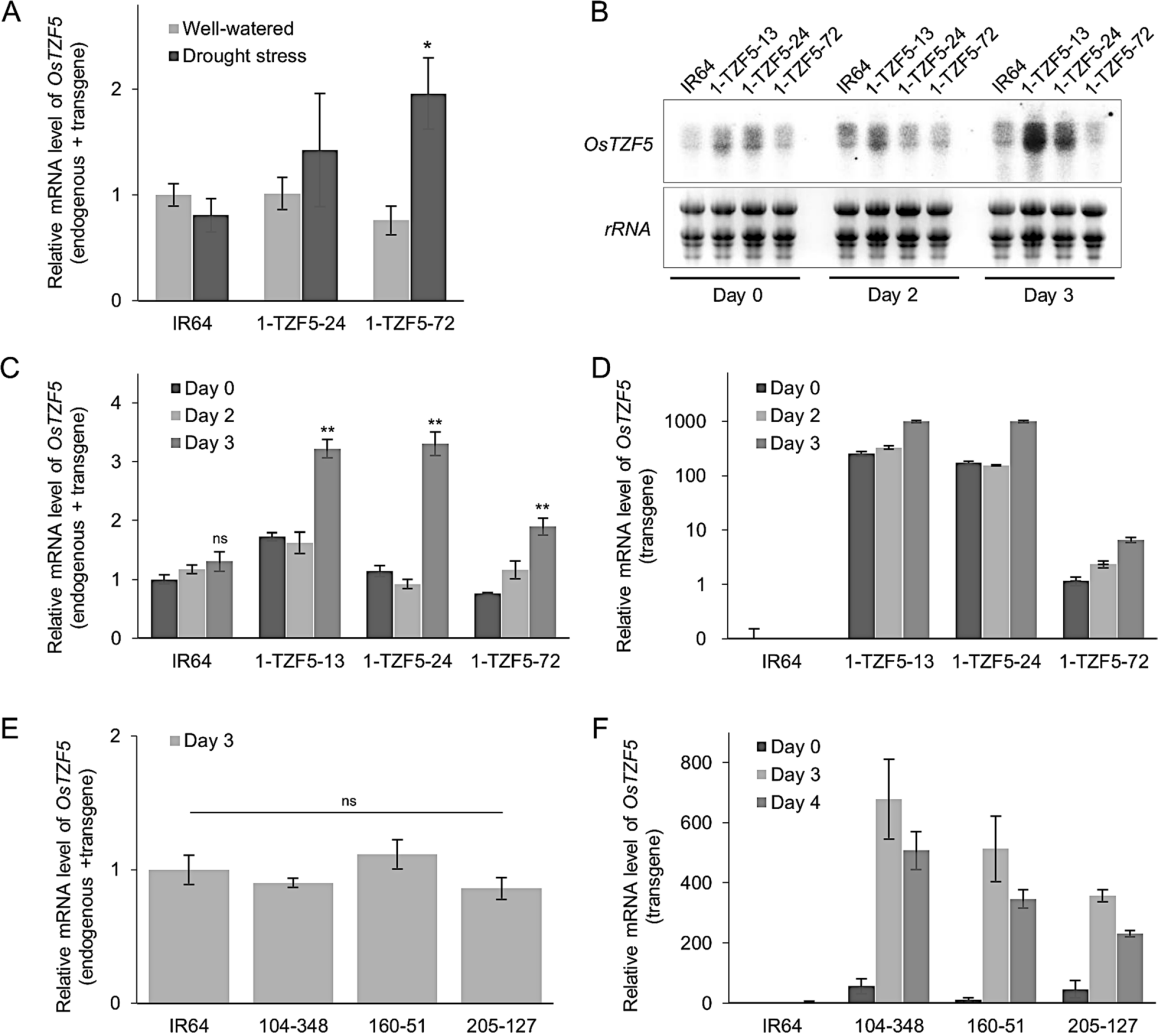
*OsTZF5* expression in the drought susceptible wild type IR64 and 1-TZF5 transgenic lines at the T_3_ and T_5_ generations and in pyramided lines at the BC_2_F_5_ generation. Transgenic lines from cylinder trial C2: **A** *OsTZF5* endogenous and transgene expression in the well-watered and drought stress treatments at 16 days after draining (38 days after sowing) using the *OsActin1* gene as reference (*p < 0.05 from well-watered conditions). Transgenic lines analyzed at JIRCAS: **B** Northern blot of *OsTZF5* expression, and real-time quantitative PCR using primers detecting **C** *OsTZF5* endogenous gene and transgene expression and** D**) transgene specific expression with *18S rRNA* gene as a reference in both cases (**p < 0.01 and ns means no significance from Day 0). Pyramiding lines: real-time quantitative PCR using primers detecting **E** *OsTZF5* endogenous gene and transgene expression and **F** transgene specific expression with *OsUbi1* gene as a reference in both cases (ns means no significance from IR64). Primers are described in Tables S6 and S7. Bars represent mean values ± se of three biological replicates

Given that the presence of the transgene was verified in each trial, the changes in drought-yield advantage over generations indicated that some genetic factors independent of the presence of the transgene were also changing over generations. To verify the overexpression of the transgene under drought as conferred by the *LIP9* promoter, and to investigate if transgene silencing occurred over generations, we measured the transcript abundance of *OsTZF5* at T_3_, T_4_, T_5_, and T_6_ generations in the transgenic lines and at the BC_2_F_5_ generation in the pyramided lines. In each case, drought stress increased *OsTZF5* expression and no decrease in expression level in the transgenics were detected across generations (Fig. 3A–F). No significant change in endogenous *OsTZF5* transcripts were detected after drought imposition in IR64 while a marked increase in *OsTZF5* expression in the three transgenic lines (2 to fourfold) were observed on day 3 (Fig. 3C). However, transgene-specific transcripts were also detected at the time of initiation of the drought stress, suggesting that the *OsTZF5* transgenes were expressed under non-drought conditions, although at lower levels. Drought stress induced transgene-specific expression was observed in the pyramided lines but not in IR64, but the total level of *OsTZF5* expression (endogenous + transgenic) was similar to that of IR64. These gene expression results indicate that the transgene was not silenced among multiple generations and seed sources.

### Genetic Background Effect on Agronomic Traits

**Fig. 4:**
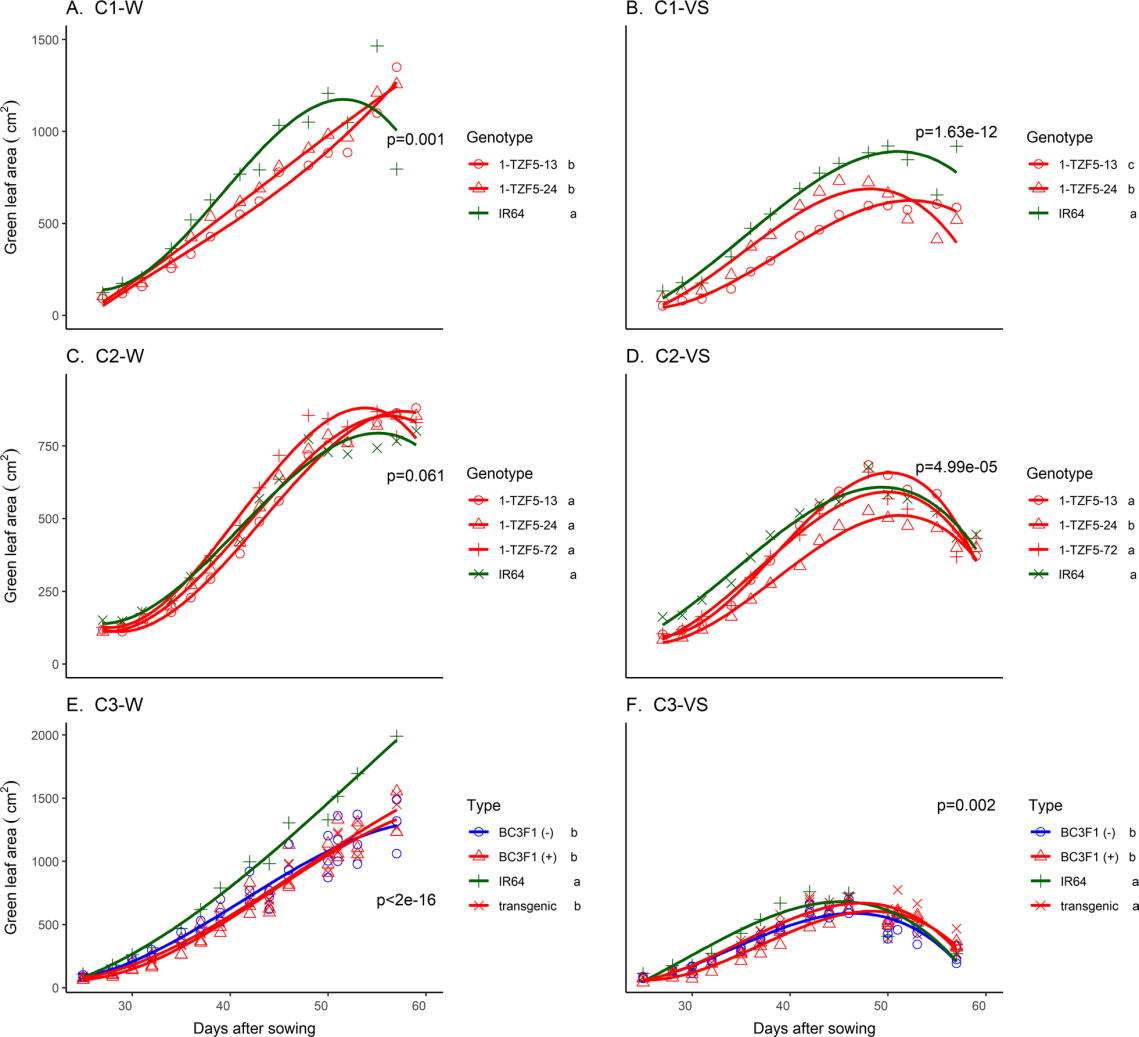
Green leaf area across cylinder studies in **A**−**D** transgenic lines and **E**–**F** the background-cleaned lines and transgenics. Symbols represent means per transgenic line or genotype group and were compared across dates by ANOVA. Significant differences among lines/genotype groups are indicated by the letters next to the legend in each panel. C: Cylinder. W: well-watered, VS: vegetative stage drought stress. IR64: drought susceptible wild-type, 1-TZF5-13, 1-TZF5-24, 1- T-TZF5-72: transgenic lines, BC3F1(−): background-cleaned lines without the transgene, BC3F1(+): background-cleaned lines with the transgene, transgenic: 1-TZF5-13, 1-TZF5-24, and 1- T-TZF5-72

To further dissect the role of the transgene in conferring drought tolerance, the background of the lead transgenic event 1-TZF5-72 was cleaned by backcrossing with IR64 three times (Additional file 2: Fig. S3). The level of polymorphism was < 1% in background cleaned lines (Additional file 2: Table S10). Although grain yield could not be assessed in the background-cleaned lines, these lines showed a similar shorter DTF and smaller aboveground biomass (as indicated by leaf area) than IR64 in both treatments of trials C3 and SH6 (Fig. 4, Additional file 2: Table S11 and Fig. S10).

The *OsTZF5* transgene was also evaluated in an IR64 genetic background containing known drought-yield QTLs to assess the potential additive effect of the transgene in a drought-tolerant rice variety. The *OsTZF5* transgene was pyramided into a major-effect drought yield QTL NIL IR87707-445-B-B-B through crossing with the lead transgenic event 1-TZF5-72 (Additional file 2: Fig. S4). An average polymorphism of 6.9% between 1-TZF5-72 and IR64, 5.9% between 1-TZF5-72 and IR87707-445-B-B-B (*qDTY*_*2.2*_ and *qDTY*_*4.1*_ regions excluded), and 1.35–1.69% between the two BC_2_F_4_ pyramided lines and IR64 were observed (Additional file 2: Table S10). In the Pyramiding trials, the grain yield advantage over IR64 was more strongly affected by individual genotype and the severity of the drought stress across trials (Additional file 2: Table S12). The mean grain yields of some transgene-QTL pyramided lines were higher than IR64 in both treatments of individual experiments, except under severe stress (SH8-RS) when no significant difference was observed among genotypes (Additional file 2: Table S12). The pyramided lines did not show the same differences with IR64 or the same generational effects as observed in the transgenic lines: grain yield, harvest index, and biomass did not show consistent trends in relation to IR64 in either treatment (Additional file 2: Fig. S11A–C). However, the time to flowering of the pyramided lines was generally shorter than that of IR64 (Additional file 2: Fig. S11D), although the time to flowering was also affected by the severity of stress, season, and genotype (Additional file 2: Table S13). The yield under well-watered conditions was lower and the time to flowering was shorter in the pyramided lines compared to the QTL NIL (Additional file 2: Table S13).

### Mechanistic Characterization of the Transgenics

We observed smaller leaf area in the transgenic lines (Trials C1 and C2) as well as in the background cleaned lines (Trial C3; Fig. 4, Additional file 2: Fig. S10) when compared with IR64 in the cylinder studies in both treatments. These differences in leaf area were reflected in slightly lower early water uptake rates of the transgenic lines as compared with IR64 in the cylinder trials (Additional file 2: Fig. S12 A, B, D). Water uptake rates were lower in the nulls compared to the transgenics in cylinder study C1 only (Additional file 2: Fig. S13).

Despite the differences in water uptake observed in the cylinder trials, in the screenhouse and field trials we observed no significant differences between the transgenic lines and IR64 for canopy temperature, stomatal conductance, photosynthesis, leaf relative water content, leaf water potential, or leaf osmotic potential under drought stress conditions (Additional file 2: Table S14, Figs. S14, S15). Likewise, no differences between the transgenic lines and IR64 were observed for root length density to a depth of 60 cm in Trials F-VRS and SH2-S (Additional file 2: Fig. S16). Most of these measurements were also performed in the well-watered treatments, in which no differences were observed among genotypes (Additional file 2: Table S14, Fig. S15). In the pyramided lines, IR 125537-104-224 showed lower canopy temperature than IR64 on certain dates across two drought experiments (SH7-RS and SH9-RS), but not under severe stress (SH8-RS) (Additional file 2: Fig. S17).

With the idea that better resource allocation may have influenced harvest index and consequently grain yield in the transgenic lines, we measured the carbohydrate accumulation in stems in the transgenic lines in Trial SH4 and in the background cleaned lines in Trial SH6. In general, the transgenic lines tended to show lower stem carbohydrate levels than IR64 at later measurement dates, except in the background cleaned lines under well-watered conditions which showed generally higher stem soluble sugar levels than IR64 at the final sampling date (Additional file 2: Figs. S18, S19).

## Discussion

In this study, we describe three transgenic lines overexpressing the *OsTZF5* transgene that showed significantly higher yield than IR64 under drought stress conditions in the early generations following in vitro transformation, but whose yield advantage gradually reduced with advancing generations. Yield advantage in the transgenic lines corresponded with early DTF, smaller shoot biomass and higher harvest index. The transgenic lines showed similar reductions with advancing generations for some of these traits as well, despite confirmation of the presence of the transgene in all experiments and observation of drought-inducible transgene expression under the *LIP9* promoter across generations.

The drought stress conditions across the lowland experiments in this study progressed relatively slowly and varied depending on atmospheric conditions, which is typical of rice lowland drought studies. This was the case for both the field and screenhouse paddy trials as well as the greenhouse cylinder and gene expression tube conditions since all container studies maintained a small plant to soil volume ratio. This is an important consideration because container size affects plant growth and small decreases in the rate of soil drying are magnified as advantages in container studies (Passioura 2006; Poorter et al. 2012; Langstroff et al. 2021). The negative reduction (e.g. increase) in biomass observed in some yield trials was likely due to the experimental setup in which the drought stress treatment was covered by a roof and the well-watered treatment was not, which resulted in some temperature differences that likely promoted vegetative growth in the drought treatment. However, the environmental conditions did not correspond to any generational effect observed.

At the morphological level, smaller shoot biomass in the transgenic and background-cleaned lines in comparison with IR64 likely led to lower water uptake rates under drought stress (Additional file 2: Fig. S12), which indirectly resulted in a water saving strategy beneficial for drought tolerance (Sinclair 2005; Lobet et al. 2014). Earlier time to flowering is generally considered as beneficial for drought tolerance by allowing the plant to escape drought stress which can be particularly detrimental at the reproductive stage (O’Toole 1982; Xu et al. 2005; Guan et al. 2010). Therefore, our results suggest that drought tolerance in the transgenics was observed when traits related to lower water consumption were affected, such as smaller biomass and earlier flowering. Interestingly, Selvaraj et al. (2020) also observed *OsTZF5* to have an effect on early flowering when over-expressed in the background on upland rice varieties Curinga and NERICA4.

Based on the various plant water status and root measurements across this study, we did not find evidence of drought avoidance traits (e.g. deeper root growth or restricted transpiration) related to the transgene. The stability of harvest index across early-generation transgenic experiments suggested that resource remobilization might be related to the yield advantage of the transgenics, and the lower late-season stem soluble sugar levels under drought (Additional file 2: Fig. S18) agree with that hypothesis, but this may equally reflect the necessity of an alternative grain-filling mechanism when drought avoidance traits are not present. Notably, the *qDTY* donor parent of our pyramiding lines has shown improved drought avoidance traits compared to IR64 (Swamy et al. 2013; Henry et al. 2015) and also high stem soluble sugar levels at harvest (Torres et al. 2020). With these distinct mechanisms of the transgenics and *qDTY* NIL, we hypothesized that pyramiding of the transgene with *qDTY*_*2.2*_ and *qDTY*_*4.1*_ could result in functional complementarity, but that was not observed in the pyramided lines tested which did not show the same degree of drought avoidance as the *qDTY* donor parent based on canopy temperature (Additional file 2: Fig. S17).

Although we cannot conclusively identify the explanatory genetic factors behind the loss of yield advantage of the transgenics under drought across generations, our results help in distinguishing among potential drought tolerance mechanisms, since early flowering and harvest index most closely reflected the levels of yield advantage in the transgenics across generations. The effect of smaller biomass, which is a common confounding factor in transgenic drought studies conducted in small pots and under severe/survival levels of stress, remained stable and did not reflect the changes in yield advantage across generations.

Several commonly-cited reasons for lack of transgene effectiveness include transgene silencing, somaclonal variation, and methylation. Transgene silencing has been reported as an important factor in the loss of transgene effect in some cases (for a review see Rajeevkumar et al. 2015) In our study, we detected gene expression under drought in early (T_3_) and advanced generations (T_5_) as well as in the pyramiding lines, indicating that gene silencing did not occur across generations. The level of *OsTZF5* overexpression varied among transgenic lines under drought stress; such variation in expression among transgenic events may be due to the pattern of transgene integration and depend on the growth stage at which stress was imposed, the stress intensity, and other genetic and environmental factors (Matzke and Matzke 1998). Reduced expression levels of the *OsTZF5* transgene in the well-watered treatment were expected due to the use of *LIP9*, a drought-inducible promoter (Nakashima et al. 2014), which was selected to avoid any possible detrimental phenotypic effects of transgene overexpression in well-watered conditions. Based on the confirmation of elevated transgene expression levels under drought in this study (Fig. 3), we conclude that transgene silencing was not responsible for the loss of yield advantage we observed with advancing generations.

By including azygous nulls in our trials on the selected transgenic lines, we noticed that nulls also showed some degree of biomass reduction and earlier flowering time as compared to IR64. These results suggested that somaclonal variation may have been related to the earlier flowering and smaller early biomass observed in the transgenic lines. Heritable somaclonal genomic mutations concomitant with epigenetic alterations can occur extensively in tissue-cultured rice (Miguel and Marum 2011). For instance, alterations in rice morphological traits (plant height, flag leaf, panicles, etc.) or physiological traits (e.g. chlorophyll fluorescence) that in some cases can induce better agronomic performance under stress have been observed after in vitro culture (Winicov 1996; Bertin et al. 1997; Van Sint et al. 1997; Lee et al. 1999; Mohan Jain 2001; Verma et al. 2013). However, it is difficult to determine if somaclonal variation was an important factor in our study because our results on the azygous nulls of the transgenic lines were limited and the biomass of the pyramided lines varied (Additional file 2: Fig. S11C) and was higher than that of IR64 in a number of trials (similar to that of the *qDTY* donor parent; Additional file 2: Table S13). Therefore, any effect on reducing biomass that was seen in the azygous nulls and background cleaned lines seemed to be lost with the presence of *qDTY*_*2.2*_ and *qDTY*_*4.1*_.

The tissue culture step of transgenic plant generation is known to induce loss of methylation on endogenous genes (Razin and Cedar 1991). Therefore, loss of DNA methylation (DNA hypomethylation) may have occurred at the T_0_ generation due to the tissue culture process, followed by re-methylation in the subsequent generations. DNA hypomethylation at promoters was associated with misregulated gene expression that may have severe impact on the plants. This severe impact may not be obvious in early generations when the plants are heterozygous, but could become apparent in advanced generations when the degree of homozygosity increases or the methylation status is reinstated to its initial levels. DNA methylation has been reported to be sufficient to influence flowering (Finnegan et al. 1998), which might explain the loss of early flowering of the late-generation transgenics in this study. Increased levels of homozygosity in the gene loci with methylation effects at promoter regions (associated with misexpression of certain protein-coding genes or transposable element) may contribute to the decreased advantage of transgenics over generations (Stroud et al. 2013). However, it is not clear why the background-cleaned lines in our study retained the effects on flowering time and biomass despite three backcrosses and similar background to IR64 (Additional file 2: Fig. S3, Table S11).

Another possible explanation for the trends observed across generations is heterosis. Heterosis is a well-known phenomenon in plant breeding causing increased vigor (Hochholdinger and Baldauf 2018). According to the dominance model, heterosis can be explained by the presence of superior dominant alleles compensating for the presence of many slightly deleterious recessive alleles (Lippman and Zamir 2007). In our study a rather detrimental effect of decreased heterozygosity on biomass (vigor) under drought was observed. The in vitro transformation may have altered the balance between deleterious and superior alleles that was restored by subsequent cycles of self-pollination. In addition, the level of homozygosity of the transgene itself tended to increase over generations (Additional file 2: Fig. S1B). Gene dosage can have significant effects on gene function without necessarily affecting gene expression (Liu et al. 2003; Krieger et al 2010).  It is possible that a certain level of transgene heterosis explains the superior performance of the early-generation transgenic lines in this study. In this hypothesis, the heterozygous state of the *OsTZF5* transgene would be superior to its homozygous state.

## Conclusions

One of the main aims of our analysis was to identify the transgene-specific physiological effects responsible for the yield increase observed in the *LIP9:OsTZF5* transgenic lines under drought stress, which declined across generations. Without the extensive characterization to which the transgenics in this study were subjected over time, these complications would not have been known. The large number of yield trials, continuation to advanced generations, and exploration of variation in the genetic background effects in this study provides a broad context that should be taken into consideration for future transgenic/drought tolerance studies. Given the promising yield under drought results observed in the early-generation transgenic lines of this study, more research is necessary to understand how beneficial effects of the genetic background can be harnessed to provide stable levels of drought tolerance across generations.

### Supplementary Information

**Additional file 1:** Information pertaining to the genes and plasmid used in this study.

**Additional ﻿file 2:** Supplemental tables and figures.

## Data Availability

Data will be made available on the IRRI Dataverse site upon publication.
