# Supplementary material for: A Case Study from the Overexpression of OsTZF5, Encoding a CCCH Tandem Zinc Finger Protein, in Rice Plants Across Nineteen Yield Trials
Source: Rice (N Y). 2024 Apr 9;17:25. doi: 10.1186/s12284-024-00705-z (PMC11003944; doi:10.1186/s12284-024-00705-z)
Supplement: Supplementary file 1 — Additional file 1: Information pertaining to the genes and plasmid used in this study. [file 12284_2024_705_MOESM1_ESM.docx]

**Additional File 1.** Information pertaining to the genes and plasmid used in this study.

**Accession no.:**

*OsLIP9* (Os02g0669100/LOC_Os02g44870)),

*OsTZF5* (Os05g0128200/LOC_Os05g03760)

**Sequence:**

**>*LIP9* promoter (1,061 bp)**

TCATCAGCTATCATCAAAGCGAAGGAAAGAAAGAAAAATAAAAGGAAAAGAACTGGCTGGAAATTAGAGAAGCCCCGGACGACTCGATCTGGGGGTGGCAAATTAATCAGTGTGATCAACAGGGATAACTTATCCCGTCCGACCAAATCCACCAACCAAACCAAGACCCGATTTGTTAGGCTGTGAAAGACGGATCAGTGGGACCCTGATCTACGGACCCCATATGTCACCGTCCAGGTCTCTGGATCTCTCCCGTCGTCCTAATCAGACACCGCGCGCGCGGTGCCGTCGCTCTCGAGCCGTGTCCCGCTCCCAACTCGTCACAAAAGCGATCACAGACTCTTCCTTCCTCTGCTGGGAGAGAAGAAAAATTGGCCGCGATGATGCCGATAAAGAGGAAAAAGGGATGAGAATCCGATGGAAAAAAACTGATGTTAATCTATCGCTACTGCTGCGCACTAAGACGAATCGTATCCGAACAAGAAACGCTTACGTTACTGTTCCTAAATGGATCGCTCCGCTCATCACTTAACCAAAAATCGATTAGGAAATTGACGGACAGCGACGCCCGAAGCCAAGTGTCTCGTCGCGTAGGCGTCGAGGCCTCGAAGCAGAGGGAGCGGAGAGGCGGACGCGCCGCCCACGCCTCCTCTCCCTCGGTGACACGGCCGTCTGGCTCCACATGGCGCCGACCTCTCCCGATGCGTCCACCCGTCCCGAGGCACCGCCACGTCGGAACCAGCCGGCCGCCCCACGCGATTGCCGACACGCGTCGCGGCGCCACTGGCTCACCCGCTGCCTGCCTCTGCCTGCCCCCCATCTCGTCGCCATTTCCCGCCCACGCTTCTTGTCCTCGCGTCGCCTACGCGTACGTACGATACAAACGCCGCACCTTTCGATCCCCTCCGCTATATAAGGAGGGCATCTGCCTCGCCACCTTCTTCATCCGAAAGCAAAAGCGACTCGTCACAGCTCAAACAAGTCAAGAGCGAATAGTTCTTGCTGATCTGTTGTTTGATTACTTTAGTTCTCGAGAGGCTTTAGCTGAATCCATCGATCGA

**>*OsTZF5* (1,806 bp)**

ATGTGCTCTGGGCCGCGCAAGCCGTCCACACCGCCGCTGCCGCAGCAGCAGAAGGAGGCGACGGTGATGGCGGCGTCCTTGCTTCTTGAGCTGGCGGCAGCGGACGACGTGGCGGCGGTGAGGAGGGTCGTGGAGGAGGAGAAGGTGTCTCTTGGCGTGGCTGGGTTGTGGTATGGGCCTTCGGCGAGCGGCGTGGCGAGGCTCGGGATGGAGCGGAGGACGGCGGCGATGGTGGCGGCGCTGTACGGGAGCACGGGGGTGCTTGGGTATGTCGTGGCGGCAGCGCCGGCGGAGGCCGCGCGCGCGTCGGAGACGGATGGGGCCACGCCGCTGCACATGGCGGCTGCCGGTGGCGCGGCGAACGCGGTCGCGGCCACGCGCCTGTTGCTCGCCGCGGGGGCGTCGGTCGACGCGCTCTCGGCTTCGGGGCTCCGCGCCGGTGACCTCCTCCCGCGCGCCACCGCGGCGGAGAAGGCCATCCGGCTGCTGCTCAAGTCGCCGGCCGTGTCGCCGTCGTCGTCGCCGAAGAAGTCGGCCTCGCCGCCGTCGCCGCCGCCGCCGCAGGAGGCGAAGAAGGAGTACCCGCCTGACCTGACGCTGCCCGACCTCAAGAGCGGACTGTTCAGCACCGACGAGTTCCGCATGTACAGCTTCAAGGTGAAGCCGTGCTCCCGCGCCTACTCCCATGACTGGACCGAGTGCCCCTTCGTCCACCCCGGCGAGAACGCGCGCCGCCGCGACCCTCGCCGCTACTCCTACAGCTGCGTGCCTTGCCCGGAGTTCCGCAAGGGCGGCTCGTGCCGCAAGGGCGACGCGTGCGAGTACGCCCATGGCGTGTTCGAGTGCTGGCTCCACCCGGCGCAGTACAGGACGCGCCTCTGCAAGGACGAGGTCGGCTGCGCGCGCCGCATCTGCTTCTTCGCCCACAAGCCCGACGAGCTCCGCGCCGTCAACCCCTCCGCCGTGTCCGTCGGCATGCAGCCCACCGTATCGTCGCCGCGCTCCTCGCCGCCCAACGGGCTCGACATGGCGGCGGCGGCGGCGGCGATGATGAGCCCCGCCTGGCCGTCGTCCCCAGCGAGCCGCCTCAAGACGGCGCTCGGCGCGCGGGAGCTCGACTTCGACCTCGAGATGCTCGCGCTGGACCAGTACCAGCAGAAGCTGTTCGACAAGGTGTCCGGCGCGCCGTCGCCGAGGGCGAGCTGGGGCGCCGCGGCGAACGGCCTCGCCACCGCGTCGCCGGCGAGGGCCGTGCCGGACTACACCGACCTGCTCGGCTCCGTCGACCCGGCCATGCTGTCCCAGCTCCACGCGCTGTCCCTCAAGCAGGCCGGCGACATGCCCGCGTACAGCTCCATGGCGGACACCACGCAGATGCACATGCCGACCTCGCCGATGGTGGGCGGCGCGAACACCGCGTTCGGGCTGGACCACTCCATGGCGAAGGCGATCATGAGCTCCCGCGCCTCGGCGTTCGCCAAGCGCAGCCAGAGCTTCATCGACCGCGGAGGCCGCGCCCCGGCGGCGCGTTCGCTCATGTCGCCGGCGACGACCGGCGCGCCGTCCATTCTCTCGGACTGGGGCTCGCCGGACGGCAAGCTGGACTGGGGCGTCCAGGGCGACGAGCTGCACAAGCTCCGCAAGTCGGCGTCGTTCGCGTTCCGCGGCCAATCCGCCATGCCGGTGGCGACGCACGCCGCGGCGGCGGAGCCGGACGTGTCATGGGTGAACTCTCTTGTCAAGGACGGCCACGCCGCCGGCGACATATTCGCGCAGTGGCCGGAGCAGGAGCAGATGGTGGCATGA

**>pBIH-lip9:OsTZF5 (14,477 bp)**

tgagcgtcgcaaaggcgctcggtcttgccttgctcgtcggtgatgtacttcaccagctccgcgaagtcgctcttcttgatggagcgcatggggacgtgcttggcaatcacgcgcaccccccggccgttttagcggctaaaaaagtcatggctctgccctcgggcggaccacgcccatcatgaccttgccaagctcgtcctgcttctcttcgatcttcgccagcagggcgaggatcgtggcatcaccgaaccgcgccgtgcgcgggtcgtcggtgagccagagtttcagcaggccgcccaggcggcccaggtcgccattgatgcgggccagctcgcggacgtgctcatagtccacgacgcccgtgattttgtagccctggccgacggccagcaggtaggccgacaggctcatgccggccgccgccgccttttcctcaatcgctcttcgttcgtctggaaggcagtacaccttgataggtgggctgcccttcctggttggcttggtttcatcagccatccgcttgccctcatctgttacgccggcggtagccggccagcctcgcagagcaggattcccgttgagcaccgccaggtgcgaataagggacagtgaagaaggaacacccgctcgcgggtgggcctacttcacctatcctgcccggctgacgccgttggatacaccaaggaaagtctacacgaaccctttggcaaaatcctgtatatcgtgcgaaaaaggatggatataccgaaaaaatcgctataatgaccccgaagcagggttatgcagcggaaaagcgccacgcttcccgaagggagaaaggcggacaggtatccggtaagcggcagggtcggaacaggagagcgcacgagggagcttccagggggaaacgcctggtatctttatagtcctgtcgggtttcgccacctctgacttgagcgtcgatttttgtgatgctcgtcaggggggcggagcctatggaaaaacgccagcaacgcggcctttttacggttcctggccttttgctggccttttgctcacatgttctttcctgcgttatcccctgattctgtggataaccgtattaccgcctttgagtgagctgataccgctcgccgcagccgaacgaccgagcgcagcgagtcagtgagcgaggaagcggaagagcgccagaaggccgccagagaggccgagcgcggccgtgaggcttggacgctagggcagggcatgaaaaagcccgtagcgggctgctacgggcgtctgacgcggtggaaagggggaggggatgttgtctacatggctctgctgtagtgagtgggttgcgctccggcagcggtcctgatcaatcgtcaccctttctcggtccttcaacgttcctgacaacgagcctccttttcgccaatccatcgacaatcaccgcgagtccctgctcgaacgctgcgtccggaccggcttcgtcgaaggcgtctatcgcggcccgcaacagcggcgagagcggagcctgttcaacggtgccgccgcgctcgccggcatcgctgtcgccggcctgctcctcaagcacggccccaacagtgaagtagctgattgtcatcagcgcattgacggcgtccccggccgaaaaacccgcctcgcagaggaagcgaagctgcgcgtcggccgtttccatctgcggtgcgcccggtcgcgtgccggcatggatgcgcgcgccatcgcggtaggcgagcagcgcctgcctgaagctgcgggcattcccgatcagaaatgagcgccagtcgtcgtcggctctcggcaccgaatgcgtatgattctccgccagcatggcttcggccagtgcgtcgagcagcgcccgcttgttcctgaagtgccagtaaagcgccggctgctgaacccccaaccgttccgccagtttgcgtgtcgtcagaccgtctacgccgacctcgttcaacaggtccagggcggcacggatcactgtattcggctgcaactttgtcatgcttgacactttatcactgataaacataatatgtccaccaacttatcagtgataaagaatccgcgcgttcaatcggaccagcggaggctggtccggaggccagacgtgaaacccaacatacccctgatcgtaattctgagcactgtcgcgctcgacgctgtcggcatcggcctgattatgccggtgctgccgggcctcctgcgcgatctggttcactcgaacgacgtcaccgcccactatggcattctgctggcgctgtatgcgttggtgcaatttgcctgcgcacctgtgctgggcgcgctgtcggatcgtttcgggcggcggccaatcttgctcgtctcgctggccggcgccagatctggggaaccctgtggttggcatgcacatacaaatggacgaacggataaaccttttcacgcccttttaaatatccgattattctaataaacgctcttttctcttaggtttacccgccaatatatcctgtcaaacactgatagtttaaactgaaggcgggaaacgacaatctgatcatgagcggagaattaagggagtcacgttatgacccccgccgatgacgcgggacaagccgttttacgtttggaactgacagaaccgcaacgttgaaggagccactcagccaattcccatcttgaaagaaatatagtttaaatatttattgataaaataacaagtcaggtattatagtccaagcaaaaacataaatttattgatgcaagtttaaattcagaaatatttcaataactgattatatcagctggtacattgccgtagatgaaagactgagtgcgatattatgtgtaatacataaattgatgatatagctagcttagctcatcgggggatcgatcccggtcggcatctactctattcctttgccctcggacgagtgctggggcgtcggtttccactatcggcgagtacttctacacagccatcggtccagacggccgcgcttctgcgggcgatttgtgtacgcccgacagtcccggctccggatcggacgattgcgtcgcatcgaccctgcgcccaagctgcatcatcgaaattgccgtcaaccaagctctgatagagttggtcaagaccaatgcggagcatatacgcccggagccgcggcgatcctgcaagctccggatgcctccgctcgaagtagcgcgtctgctgctccatacaagccaaccacggcctccagaagaagatgttggcgacctcgtattgggaatccccgaacatcgcctcgctccagtcaatgaccgctgttatgcggccattgtccgtcaggacattgttggagccgaaatccgcgtgcacgaggtgccggacttcggggcagtcctcggcccaaagcatcagctcatcgagagcctgcgcgacggacgcactgacggtgtcgtccatcacagtttgccagtgatacacatggggatcagcaatcgcgcatatgaaatcacgccatgtagtgtattgaccgattccttgcggtccgaatgggccgaacccgctcgtctggctaagatcggccgcagcgatcgcatccatggcctccgcgaccggctgcagaacagcgggcagttcggtttcaggcaggtcttgcaacgtgacaccctgtgcacggcgggagatgcaataggtcaggctctcgctgaatgccccaatgtcaagcacttccggaatcgggagcgcggccgatgcaaagtgccgataaacataacgatctttgtagaaaccatcggcgcagctatttacccgcaggacatatccacgccctcctacatcgaagctgaaagcacgagattcttcgccctccgagagctgcatcaggtcggagacgctgtcgaacttttcgatcagaaacttctcgacagacgtcgcggtgagttcaggctttttcatatcttattgcccccctagagtcgagatctggattgagagtgaatatgagactctaattggataccgaggggaatttatggaacgtcagtggagcatttttgacaagaaatatttgctagctgatagtgaccttaggcgacttttgaacgcgcaataatggtttctgacgtatgtgcttagctcattaaactccagaaacccgcggctgagtggctccttcaacgttgcggttctgtcagttccaaacgtaaaacggcttgtcccgcgtcatcggcgggggtcataacgtgactcccttaattctccgctcatgatcttgatcccctgcgccatcagatccttggcggcaagaaagccatccagtttactttgcagggcttcccaaccttaccagagggcgccccagctggcaattccggttcgcttgctgtccataaaaccgcccagtctagctatcgccatgtaagcccactgcaagctacctgctttctctttgcgcttgcgttttcccttgtccagatagcccagtagctgacattcatccggggtcagcaccgtttctgcggactggctttctacgtgttccgcttcctttagcagcccttgcgccctgagtgcttgcggcagcgtgAAGCTTTCATCAGCTATCATCAAAGCGAAGGAAAGAAAGAAAAATAAAAGGAAAAGAACTGGCTGGAAATTAGAGAAGCCCCGGACGACTCGATCTGGGGGTGGCAAATTAATCAGTGTGATCAACAGGGATAACTTATCCCGTCCGACCAAATCCACCAACCAAACCAAGACCCGATTTGTTAGGCTGTGAAAGACGGATCAGTGGGACCCTGATCTACGGACCCCATATGTCACCGTCCAGGTCTCTGGATCTCTCCCGTCGTCCTAATCAGACACCGCGCGCGCGGTGCCGTCGCTCTCGAGCCGTGTCCCGCTCCCAACTCGTCACAAAAGCGATCACAGACTCTTCCTTCCTCTGCTGGGAGAGAAGAAAAATTGGCCGCGATGATGCCGATAAAGAGGAAAAAGGGATGAGAATCCGATGGAAAAAAACTGATGTTAATCTATCGCTACTGCTGCGCACTAAGACGAATCGTATCCGAACAAGAAACGCTTACGTTACTGTTCCTAAATGGATCGCTCCGCTCATCACTTAACCAAAAATCGATTAGGAAATTGACGGACAGCGACGCCCGAAGCCAAGTGTCTCGTCGCGTAGGCGTCGAGGCCTCGAAGCAGAGGGAGCGGAGAGGCGGACGCGCCGCCCACGCCTCCTCTCCCTCGGTGACACGGCCGTCTGGCTCCACATGGCGCCGACCTCTCCCGATGCGTCCACCCGTCCCGAGGCACCGCCACGTCGGAACCAGCCGGCCGCCCCACGCGATTGCCGACACGCGTCGCGGCGCCACTGGCTCACCCGCTGCCTGCCTCTGCCTGCCCCCCATCTCGTCGCCATTTCCCGCCCACGCTTCTTGTCCTCGCGTCGCCTACGCGTACGTACGATACAAACGCCGCACCTTTCGATCCCCTCCGCTATATAAGGAGGGCATCTGCCTCGCCACCTTCTTCATCCGAAAGCAAAAGCGACTCGTCACAGCTCAAACAAGTCAAGAGCGAATAGTTCTTGCTGATCTGTTGTTTGATTACTTTAGTTCTCGAGAGGCTTTAGCTGAATCCATCGATCGAGGATCCCCGGGATGTGCTCTGGGCCGCGCAAGCCGTCCACACCGCCGCTGCCGCAGCAGCAGAAGGAGGCGACGGTGATGGCGGCGTCCTTGCTTCTTGAGCTGGCGGCAGCGGACGACGTGGCGGCGGTGAGGAGGGTCGTGGAGGAGGAGAAGGTGTCTCTTGGCGTGGCTGGGTTGTGGTATGGGCCTTCGGCGAGCGGCGTGGCGAGGCTCGGGATGGAGCGGAGGACGGCGGCGATGGTGGCGGCGCTGTACGGGAGCACGGGGGTGCTTGGGTATGTCGTGGCGGCAGCGCCGGCGGAGGCCGCGCGCGCGTCGGAGACGGATGGGGCCACGCCGCTGCACATGGCGGCTGCCGGTGGCGCGGCGAACGCGGTCGCGGCCACGCGCCTGTTGCTCGCCGCGGGGGCGTCGGTCGACGCGCTCTCGGCTTCGGGGCTCCGCGCCGGTGACCTCCTCCCGCGCGCCACCGCGGCGGAGAAGGCCATCCGGCTGCTGCTCAAGTCGCCGGCCGTGTCGCCGTCGTCGTCGCCGAAGAAGTCGGCCTCGCCGCCGTCGCCGCCGCCGCCGCAGGAGGCGAAGAAGGAGTACCCGCCTGACCTGACGCTGCCCGACCTCAAGAGCGGACTGTTCAGCACCGACGAGTTCCGCATGTACAGCTTCAAGGTGAAGCCGTGCTCCCGCGCCTACTCCCATGACTGGACCGAGTGCCCCTTCGTCCACCCCGGCGAGAACGCGCGCCGCCGCGACCCTCGCCGCTACTCCTACAGCTGCGTGCCTTGCCCGGAGTTCCGCAAGGGCGGCTCGTGCCGCAAGGGCGACGCGTGCGAGTACGCCCATGGCGTGTTCGAGTGCTGGCTCCACCCGGCGCAGTACAGGACGCGCCTCTGCAAGGACGAGGTCGGCTGCGCGCGCCGCATCTGCTTCTTCGCCCACAAGCCCGACGAGCTCCGCGCCGTCAACCCCTCCGCCGTGTCCGTCGGCATGCAGCCCACCGTATCGTCGCCGCGCTCCTCGCCGCCCAACGGGCTCGACATGGCGGCGGCGGCGGCGGCGATGATGAGCCCCGCCTGGCCGTCGTCCCCAGCGAGCCGCCTCAAGACGGCGCTCGGCGCGCGGGAGCTCGACTTCGACCTCGAGATGCTCGCGCTGGACCAGTACCAGCAGAAGCTGTTCGACAAGGTGTCCGGCGCGCCGTCGCCGAGGGCGAGCTGGGGCGCCGCGGCGAACGGCCTCGCCACCGCGTCGCCGGCGAGGGCCGTGCCGGACTACACCGACCTGCTCGGCTCCGTCGACCCGGCCATGCTGTCCCAGCTCCACGCGCTGTCCCTCAAGCAGGCCGGCGACATGCCCGCGTACAGCTCCATGGCGGACACCACGCAGATGCACATGCCGACCTCGCCGATGGTGGGCGGCGCGAACACCGCGTTCGGGCTGGACCACTCCATGGCGAAGGCGATCATGAGCTCCCGCGCCTCGGCGTTCGCCAAGCGCAGCCAGAGCTTCATCGACCGCGGAGGCCGCGCCCCGGCGGCGCGTTCGCTCATGTCGCCGGCGACGACCGGCGCGCCGTCCATTCTCTCGGACTGGGGCTCGCCGGACGGCAAGCTGGACTGGGGCGTCCAGGGCGACGAGCTGCACAAGCTCCGCAAGTCGGCGTCGTTCGCGTTCCGCGGCCAATCCGCCATGCCGGTGGCGACGCACGCCGCGGCGGCGGAGCCGGACGTGTCATGGGTGAACTCTCTTGTCAAGGACGGCCACGCCGCCGGCGACATATTCGCGCAGTGGCCGGAGCAGGAGCAGATGGTGGCATGAGGGGCGGCCGCgagctcGAATTTCCccgatcgttcaaacatttggcaataaagtttcttaagattgaatcctgttgccggtcttgcgatgattatcatataatttctgttgaattacgttaagcatgtaataattaacatgtaatgcatgacgttatttatgagatgggtttttatgattagagtcccgcaattatacatttaatacgcgatagaaaacaaaatatagcgcgcaaactaggataaattatcgcgcgcggtgtcatctatgttactagatcgggaattcactggccgtcgttttacaacgtcgtgactgggaaaaccctggcgttacccaacttaatcgccttgcagcacatccccctttcgccagctggcgtaatagcgaagaggcccgcaccgatcgcccttcccaacagttgcgcagcctgaatggcgcccgctcctttcgctttcttcccttcctttctcgccacgttcgccggctttccccgtcaagctctaaatcgggggctccctttagggttccgatttagtgctttacggcacctcgaccccaaaaaacttgatttgggtgatggttcacgtagtgggccatcgccctgatagacggtttttcgccctttgacgttggagtccacgttctttaatagtggactcttgttccaaactggaacaacactcaaccctatctcgggctattcttttgatttataagggattttgccgatttcggaaccaccatcaaacaggattttcgcctgctggggcaaaccagcgtggaccgcttgctgcaactctctcagggccaggcggtgaagggcaatcagctgttgcccgtctcactggtgaaaagaaaaaccaccccagtacattaaaaacgtccgcaatgtgttattaagttgtctaagcgtcaatttgtttacaccacaatatatcctgccaccagccagccaacagctccccgaccggcagctcggcacaaaatcaccactcgatacaggcagcccatcagtccgggacggcgtcagcgggagagccgttgtaaggcggcagactttgctcatgttaccgatgctattcggaagaacggcaactaagctgccgggtttgaaacacggatgatctcgcggagggtagcatgttgattgtaacgatgacagagcgttgctgcctgtgatcaaatatcatctccctcgcagagatccgaattatcagccttcttattcatttctcgcttaaccgtgacaggctgtcgatcttgagaactatgccgacataataggaaatcgctggataaagccgctgaggaagctgagtggcgctatttctttagaagtgaacgttgacgatatcaactcccctatccattgctcaccgaatggtacaggtcggggacccgaagttccgactgtcggcctgatgcatccccggctgatcgaccccagatctggggctgagaaagcccagtaaggaaacaactgtaggttcgagtcgcgagatcccccggaaccaaaggaagtaggttaaacccgctccgatcaggccgagccacgccaggccgagaacattggttcctgtaggcatcgggattggcggatcaaacactaaagctactggaacgagcagaagtcctccggccgccagttgccaggcggtaaaggtgagcagaggcacgggaggttgccacttgcgggtcagcacggttccgaacgccatggaaaccgcccccgccaggcccgctgcgacgccgacaggatctagcgctgcgtttggtgtcaacaccaacagcgccacgcccgcagttccgcaaatagcccccaggaccgccatcaatcgtatcgggctacctagcagagcggcagagatgaacacgaccatcagcggctgcacagcgcctaccgtcgccgcgaccccgcccggcaggcggtagaccgaaataaacaacaagctccagaatagcgaaatattaagtgcgccgaggatgaagatgcgcatccaccagattcccgttggaatctgtcggacgatcatcacgagcaataaacccgccggcaacgcccgcagcagcataccggcgacccctcggcctcgctgttcgggctccacgaaaacgccggacagatgcgccttgtgagcgtccttggggccgtcctcctgtttgaagaccgacagcccaatgatctcgccgtcgatgtaggcgccgaatgccacggcatctcgcaaccgttcagcgaacgcctccatgggctttttctcctcgtgctcgtaaacggacccgaacatctctggagctttcttcagggccgacaatcggatctcgcggaaatcctgcacgtcggccgctccaagccgtcgaatctgagccttaatcacaattgtcaattttaatcctctgtttatcggcagttcgtagagcgcgccgtgcgtcccgagcgatactgagcgaagcaagtgcgtcgagcagtgcccgcttgttcctgaaatgccagtaaagcgctggctgctgaacccccagccggaactgaccccacaaggccctagcgtttgcaatgcaccaggtcatcattgacccaggcgtgttccaccaggccgctgcctcgcaactcttcgcaggcttcgccgacctgctcgcgccacttcttcacgcgggtggaatccgatccgcacatgaggcggaaggtttccagcttgagcgggtacggctcccggtgcgagctgaaatagtcgaacatccgtcgggccgtcggcgacagcttgcggtacttctcccatatgaatttcgtgtagtggtcgccagcaaacagcacgacgatttcctcgtcgatcaggacctggcaacgggacgttttcttgccacggtccaggacgcggaagcggtgcagcagcgacaccgattccaggtgcccaacgcggtcggacgtgaagcccatcgccgtcgcctgtaggcgcgacaggcattcctcggccttcgtgtaataccggccattgatcgaccagcccaggtcctggcaaagctcgtagaacgtgaaggtgatcggctcgccgataggggtgcgcttcgcgtactccaacacctgctgccacaccagttcgtcatcgtcggcccgcagctcgacgccggtgtaggtgatcttcacgtccttgttgacgtggaaaatgaccttgttttgcagcgcctcgcgcgggattttcttgttgcgcgtggtgaacagggcagagcgggccgtgtcgtttggcatcgctcgcatcgtgtccggccacggcgcaatatcgaacaaggaaagctgcatttccttgatctgctgcttcgtgtgtttcagcaacgcggcctgcttggcctcgctgacctgttttgccaggtcctcgccggcggtttttcgcttcttggtcgtcatagttcctcgcgtgtcgatggtcatcgacttcgccaaacctgccgcctcctgttcgagacgacgcgaacgctccacggcggccgatggcgcgggcagggcagggggagccagttgcacgctgtcgcgctcgatcttggccgtagcttgctggaccatcgagccgacggactggaaggtttcgcggggcgcacgcatgacggtgcggcttgcgatggtttcggcatcctcggcggaaaaccccgcgtcgatcagttcttgcctgtatgccttccggtcaaacgtccgattcattcaccctccttgcgggattgccccgactcacgccggggcaatgtgcccttattcctgatttgacccgcctggtgccttggtgtccagataatccaccttatcggcaatgaagtcggtcccgtagaccgtctggccgtccttctcgtacttggtattccgaatcttgccctgcacgaataccagcgaccccttgcccaaatacttgccgtgggcctcggcctgagagccaaaacacttgatgcggaagaagtcggtgcgctcctgcttgtcgccggcatcgttgcgccacatctaggtactaaaacaattcatccagtaaaatataatattttattttctcccaatcaggcttgatccccagtaagtcaaaaaatagctcgacatactgttcttccccgatatcctccctgatcgaccggacgcagaaggcaatgtcataccacttgtccgccctgccgcttctcccaagatcaataaagccacttactttgccatctttcacaaagatgttgctgtctcccaggtcgccgtgggaaaagacaagttcctcttcgggcttttccgtctttaaaaaatcatacagctcgcgcggatctttaaatggagtgtcttcttcccagttttcgcaatccacatcggccagatcgttattcagtaagtaatccaattcggctaagcggctgtctaagctattcgtatagggacaatccgatatgtcgatggagtgaaagagcctgatgcactccgcatacagctcgataatcttttcagggctttgttcatcttcatactcttccgagcaaaggacgccatcggcctcactcatgagcagattgctccagccatcatgccgttcaaagtgcaggacctttggaacaggcagctttccttccagccatagcatcatgtccttttcccgttccacatcataggtggtccctttataccggctgtccgtcatttttaaatataggttttcattttctcccaccagcttatataccttagcaggagacattccttccgtatcttttacgcagcggtatttttcgatcagttttttcaattccggtgatattctcattttagccatttattatttccttcctcttttctacagtatttaaagataccccaagaagctaattataacaagacgaactccaattcactgttccttgcattctaaaaccttaaataccagaaaacagctttttcaaagttgttttcaaagttggcgtataacatagtatcgacggagccgattttgaaaccacaattatgggtgatgctgccaacttactgatttagtgtatgatggtgtttttgaggtgctccagtggcttctgtgtctatcagctgtccctcctgttcagctactgacggggtggtgcgtaacggcaaaagcaccgccggacatcagcgctatctctgctctcactgccgtaaaacatggcaactgcagttcacttacaccgcttctcaacccggtacgcaccagaaaatcattgatatggccatgaatggcgttggatgccgggcaacagcccgcattatgggcgttggcctcaacacgattttacgtcacttaaaaaactcaggccgcagtcggtaacctcgcgcatacagccgggcagtgacgtcatcgtctgcgcggaaatggacgaacagtggggctatgtcggggctaaatcgcgccagcgctggctgttttacgcgtatgacagtctccggaagacggttgttgcgcacgtattcggtgaacgcactatggcgacgctggggcgtcttatgagcctgctgtcaccctttgacgtggtgatatggatgacggatggctggccgctgtatgaatcccgcctgaagggaaagctgcacgtaatcagcaagcgatatacgcagcgaattgagcggcataacctgaatctgaggcagcacctggcacggctgggacggaagtcgctgtcgttctcaaaatcggtggagctgcatgacaaagtcatcgggcattatctgaacataaaacactatcaataagttggagtcattacccaattatgatagaatttacaagctataaggttattgtcctgggtttcaagcattagtccatgcaagtttttatgctttgcccattctatagatatattgataagcgcgctgcctatgccttgccccctgaaatccttacatacggcgatatcttctatataaaagatatattatcttatcagtattgtcaatatattcaaggcaatctgcctcctcatcctcttcatcctcttcgtcttggtagctttttaaatatggcgcttcatagagtaattctgtaaaggtccaattctcgttttcatacctcggtataatcttacctatcacctcaaatggttcgctgggtttatcgcacccccgaacacgagcacggcacccgcgaccactatgccaagaatgcccaaggtaaaaattgccggccccgccatgaagtccgtgaatgccccgacggccgaagtgaagggcaggccgccacccaggccgccgccctcactgcccggcacctggtcgctgaatgtcgatgccagcacctgcggcacgtcaatgcttccgggcgtcgcgctcgggctgatcgcccatcccgttactgccccgatcccggcaatggcaaggactgccagcgctgccatttttggggtgaggccgttcgcggccgaggggcgcagcccctggggggatgggaggcccgcgttagcgggccgggagggttcgagaagggggggcaccccccttcggcgtgcgcggtcacgcgcacagggcgcagccctggttaaaaacaaggtttataaatattggtttaaaagcaggttaaaagacaggttagcggtggccgaaaaacgggcggaaacccttgcaaatgctggattttctgcctgtggacagcccctcaaatgtcaataggtgcgcccctcatctgtcagcactctgcccctcaagtgtcaaggatcgcgcccctcatctgtcagtagtcgcgcccctcaagtgtcaataccgcagggcacttatccccaggcttgtccacatcatctgtgggaaactcgcgtaaaatcaggcgttttcgccgatttgcgaggctggccagctccacgtcgccggccgaaatcgagcctgcccctcatctgtcaacgccgcgccgggtgagtcggcccctcaagtgtcaacgtccgcccctcatctgtcagtgagggccaagttttccgcgaggtatccacaacgccggcggccgcggtgtctcgcacacggcttcgacggcgtttctggcgcgtttgcagggccatagacggccgccagcccagcggcgagggcaaccagcccgg

Features:

2454-2478: the right border sequence

2632-2857: gene 7 terminator

2878-3903: HPT gene

3932-4219: NOS promoter

4535-4540: HindIII (AAGCTT)

4536-5601: Rice *LIP9* promoter

5602-5607: BamHI (GGATCC)

5607-5612: SmaI (CCCGGG)

5613-7418: *OsTZF5* ORF

7422-7429: NotI (GCGGCCGC)

7444-7696: NOS terminator

7701-7706: EcoRI (GAATTC)

8340-8365: the left border sequence

- *LIP9* promoter was described in Nakashima *et al.* (2007).
- *OsTZF5* was described in Selvaraj *et al.* (2020).
- Kanamycine resistance in bacteria and hygromycin resistance in plants are used.
- All DNA sequences except the *lip9* promoter and *OsTZF5* ORF sequences were derived from pBIG-HYG, a derivative of pBI121 (Becker, 1990).

**References:**

Becker D Binary vectors which allow the exchange of plant selectable markers and reporter genes. Nucleic Acids Res. (1990) 18: 203. doi: 10.1093/nar/18.1.203

Nakashima K, Tran LS, Van Nguyen D, Fujita M, Maruyama K, Todaka D, Ito Y, Hayashi N, Shinozaki K, Yamaguchi-Shinozaki K. Functional analysis of a NAC-type transcription factor OsNAC6 involved in abiotic and biotic stress-responsive gene expression in rice. Plant J. (2007) 51(4):617-30. doi: 10.1111/j.1365-313X.2007.03168.x.

Selvaraj MG, Jan A, Ishizaki T, Valencia M, Dedicova B, Maruyama K, Ogata T, Todaka D, Yamaguchi-Shinozaki K, Nakashima K, Ishitani M. Expression of the CCCH-tandem zinc finger protein gene OsTZF5 under a stress-inducible promoter mitigates the effect of drought stress on rice grain yield under field conditions. Plant Biotechnol J. (2020) 18(8):1711-1721. doi: 10.1111/pbi.13334
