# Supplementary material for: A Case Study from the Overexpression of OsTZF5, Encoding a CCCH Tandem Zinc Finger Protein, in Rice Plants Across Nineteen Yield Trials
Source: Rice (N Y). 2024 Apr 9;17:25. doi: 10.1186/s12284-024-00705-z (PMC11003944; doi:10.1186/s12284-024-00705-z)
Supplement: Supplementary file 2 — Additional ﻿file 2: Supplemental tables and figures. [file 12284_2024_705_MOESM2_ESM.docx]

**Additional file 2.** Supplemental tables and figures.

| **Table S1.** Primer pairs used for genotyping PCR analysis. | | | | |
| --- | --- | --- | --- | --- |
| Name |  | Sequence (5’ to 3’) |  | Use |
| 013A-LP1 |  | GACCAAGCTTTGTGGAGAAC |  | 1-TZF5-13 allele 1 |
| 013A-RP1 |  | GGCCAAAGTCTACTGTTGGT |  |  |
| 013A-LP2 |  | ACGGGAACTGTCCAGCATTG |  | 1-TZF5-13 allele 2 |
| 013A-RP2 |  | GCATCGATGACATGTGGGAT |  |  |
| 024A-LP1 |  | TTCTGCGTGGCACTGACAGT |  | 1-TZF5-24 allele |
| 024A-RP1 |  | AACTGACCAGGGACCTGTTC |  |  |
| 072A-LP1 |  | TGCCTGCCTGCCTAGGCAAC |  | 1-TZF5-72 allele |
| 072A-RP1 |  | TCTTATGTACCCGTGATTGC |  |  |
| F1 |  | GACGAGCTGCACAAGCTC |  | Primers within the T-DNA region |
| F2 |  | CCCTTGCGCCCTGAGTGCT |  |  |
| HPT-R (F3) |  | GCATGCAAAGTGCCGATAAA |  |  |

**Table S2.** Primers used to check for the presence of the transgene during lip9::OsTZF5-13, lip9::OsTZF5-24, and lip9::OsTZF5-72 background cleaning

| **Primer name** | **Sequence (5′to 3′)** | **Computed annealing temp. (°C)** | **Expected product size (bp)** |
| --- | --- | --- | --- |
| ZD-482-013A C2-P2 | GGT CAT TCA TCA CAT CCA G |  |  |
| ZD-482-013A C2-P3 | GAG TTC AAG TAG TCA ACA GC | 61 | **300 –Wt allele amplified** |
| ZD-482-024A -P1 | ACT TAA TCG CCT TGC AGC AC | 60 | **900- 024A allele amplified** |
| ZD-482-024A -P2 | GGA GGA AGT CAA TTC GTT GG |  |  |
| ZD-482-024A -P3 | CTT GTT GGC TTG TTG CTG TC | 60 | **550 – WT allele amplified** |
| IRNo.1_AC103981-LP1 | TGC CTG CCT GCC TAG GCA AC | 60 | 1800 WT allele amplified |
| IRNo.1_AC103981-RP1 | TCA TGA TCA GAT TGT CGT TTC C | 60 | 900 072A allele amplified |
| OsUbi1-F5 | GGA CAC AAT GAT TAG GGA TCA | 55-60 | Internal control primer |
| OsUbi-R | GTG GTG GCC AGT AAG TCC TC |  |  |

**Table S3.** Primers used to check for the presence of each transgene/QTL being introgressed into IR64 from a IR87707-445-B-B-B and lip9::OsTZF5-72 cross.

|  | | |  |
| --- | --- | --- | --- |
| Genotype | Primer used | Primer sequence | Source |
| qDTY2.2 | RM236 F | GCG CTG GTG GAA AAT GAG | Gramene SSR database |
|  | RM236 R | GGC ATC CCT CTT TGA TTC CTC |  |
| qDTY4.1 | RM518 F | CTC TTC ACT CAC TCA CCA TGG |  |
|  | RM518 R | ATC CAT CTG GAG CAA GCA AC |  |
| lip9:OsTZF5-072 | IRno.1_AC103981-RP1 | TCA TGA TCA GAT TGT CGT TTC C | JIRCAS |
|  | IRno.1_AC103981 -LP1 | TGC CTG CCT GCC TAG GCA AC |  |
| Internal control primer for the PCR reactions and its validity | OsUbi1-F5 | GGA CAC AAT GATTAG GGA TCA |  |
|  | OsUbi1- R | GTG GTG GCC AGT AAG TCC TC |  |

| **Table S4**. Pyramiding lines - Genotyping results and the number of seeds/plants obtained from each cross. | | | | |
| --- | --- | --- | --- | --- |
|  | F_1_ | BC_1_F_1_ | BC_2_F_1_ | BC_2_F_2_ |
| # of seeds obtained | 1389 | 1174 | 865 | 6932 |
| # of seeds PCR | 350 | 500 | 750 | 3700 |
| # of seeds germinated | 230 | 430 | 561 | 2580 |
| # of seeds ungerminated | 120 | 52 | 255 | 0 |
| # of plants with no amplification | 22 | 22 | 76 | 16 |
| # of plants (-) lip9:OsTZF5-72 | 101 | 230 | 353 | 16 |
| # of plants (+) lip9:OsTZF5-72 | 107 | 178 | 132 | 89 |
| # of plants with both qDTY2.2 and qDTY4.1 | - | 61 | 9 | 23 |
| # of plants with qDTY2.2 only | 0 | 34 | 28 | 10 |
| # of plants with qDTY4.1 only | 0 | 23 | 29 | 8 |
| “-“: not tested |  |  |  |  |

| **Table S5.** Primer pairs used for TAIL-PCR analysis. | | | | |
| --- | --- | --- | --- | --- |
| Name |  | Sequence (5’ to 3’) |  | Use |
| TAIL-PL1 |  | TTTCGCCTGCTGGGGCAAACCAG |  | Specific primer (SP) 1 to the left border |
| TAIL-PL2 |  | ACCGCTTGCTGCAACTCTCTCAG |  | Specific primer (SP) 2 to the left border |
| TAIL-PL3 |  | AGCTGTTGCCCGTCTCACTGGTG |  | Specific primer (SP) 3 to the left border |
| TAIL-PR1 |  | ACACAAATCGCCCGCAGAAGCGC |  | Specific primer (SP) 1 to the right border |
| TAIL-PR2 |  | CGATGGCTGTGTAGAAGTACTCGCC |  | Specific primer (SP) 2 to the right border |
| TAIL-PR3 |  | TCATGATCAGATTGTCGTTTCC |  | Specific primer (SP) 3 to the right border |
| TAIL-AD1 |  | NGTCGA(G/C)(A/T)GANA(A/T)GAA |  | Arbitary degenerate (AD) primer 1 |
| TAIL-AD2 |  | GTNCGA(G/C)(A/T)CANA(A/T)GTT |  | Arbitary degenerate (AD) primer 2 |
| TAIL-AD3 |  | (A/T)GTGNAG(A/T)ANCANAGA |  | Arbitary degenerate (AD) primer 3 |

| **Table S6.** Primer pairs used for RT-qPCR analysis. | | |  |
| --- | --- | --- | --- |
| Name | Sequence (5’ to 3’) | Use | |
| OsTZF5-171-L | CGTCCATTCTCTCGGACTGG | *OsTZF5* transgene + endogenous (transgenic lines in trial C2) | |
| OsTZF5-171-R | AGTTCACCCATGACACGTCC |  |  |
| OsTZF5-F6 | GACACCACGCAGATGCAC | *OsTZF5* transgene + endogenous (transgenic and pyramided lines analyzed at JIRCAS) | |
| OsTZF5-R1445 | TTCGCCATGGAGTGGTCCAGCCCGAAC |  |  |
| OsTZF5-F1713 | GGACGTGTCATGGGTGAACT | *OsTZF5* transgene (transgenic and pyramided lines analyzed at JIRCAS) | |
| NOSTR50 | AGACCGGCAACAGGATTCAA |  |  |
| OsAct1-L | GGTAACATTGTGCTCAGTGGTGG | *Actin1* gene | |
| OsAct1-R | AACGACCTTAATCTTCATGCTGC |  |  |
| 18SrRNA-L | AAACGGCTACCACATCCAAG | *18S rRNA* gene | |
| 18SrRNA-R | CCTCCAATGGATCCTCGTTA |  |  |
| OsUbi1-F5 | GGA CAC AAT GATTAG GGA TCA | *OsUbi1* | |
| OsUbi1- R | GTG GTG GCC AGT AAG TCC TC |  | |

| **Table S7.** Primer pairs used for northern probe preparation. | | | | |
| --- | --- | --- | --- | --- |
| Name | Sequence (5’ to 3’) | | Use | |
| AK105767-5/n | | GGGATGTGCTCTGGGCCGCG | | Full length of CDS of *OsTZF5* was used for the northern probe. |
| AK105767-3/RD/c | | TGCCACCATCTGCTCCTGCT | |  |

| **Traits** | **Line** | **Trials** |  |  |  |  |  |  |  |  |
| --- | --- | --- | --- | --- | --- | --- | --- | --- | --- | --- |
|  |  | **SH1-W** | **F-W** | **SH2-W** | **SH3-W** | **SH1-VRS** | **F-VRS** | **F-RS** | **SH2-VRS** | **SH3-VRS** |
|  |  |  |  |  |  |  |  |  |  |  |
| Grain yield (g m^-2^) | IR64 | 225 ± 23a | 384 ± 33ab | 466 ± 31b | 446 ± 96ab | 12 ± 4b | 1b | 64 ± 14b | 27 ± 14b | 25 ± 12b |
|  | 0-TZF5-13 | **-** | **-** | **-** | 262 ± 74b | **-** | **-** | **-** | **-** | 81 ± 12a |
|  | 0-TZF5-24 | **-** | **-** | **-** | 339 ± 42ab | **-** | 8 ± 4b | **-** | **-** | 25 ± 9b |
|  | 0-TZF5-72 | **-** | **-** | **-** | 287 ± 88b | **-** | **-** | **-** | **-** | 48 ± 13ab |
|  | 1-TZF5-13 | 123 ± 27b | 300 ± 16b | **-** | 306 ± 37ab | 98 ± 9a | 10 ± 5b | 212 ± 25a | **-** | 20 ± 7b |
|  | 1-TZF5-24 | 142 ± 29ab | 400 ± 43ab | **-** | 350 ± 84ab | 96 ± 14a | 11 ± 7b | 183 ± 34a | **-** | 37 ± 14b |
|  | 1-TZF5-72 | 216 ± 43ab | 362 ± 24ab | 409 ± 34b | 357 ± 48ab | 68 ± 18a | 68 ± 30a | 172 ± 24a | 70 ± 14ab | 41 ± 16b |
|  | 14-1-2-10 BIL | **-** | 469 ± 65a | 559 ± 5a | 509 ± 61a | **-** | 84 ± 27a | 233 ± 34a | 91 ± 13a | 28 ± 6b |
|  |  |  |  |  |  |  |  |  |  |  |
| Time to flowering (das) | IR64 | 85a | 79a | 68 ± 1a | 78 ± 1a | 84 ± 1a | 87a | 83 ± 1a | 69 ± 1a | 81 ± 1a |
|  | 0-TZF5-13 | - | - | - | 72 ± 1d | - | - | - | - | 72 ± 2c |
|  | 0-TZF5-24 | - | - | - | 76ab | - | 80 ± 2b | - | - | 77 ± 1ab |
|  | 0-TZF5-72 | - | - | - | 75 ± 1bc | - | - | - | - | 73 ± 1c |
|  | 1-TZF5-13 | 78 ± 1b | 74 ± 1b | - | 73 ± 2cd | 76 ± 1b | 76 ± 1bc | 76 ± 1b | - | 78 ± 3ab |
|  | 1-TZF5-24 | 76 ± 1b | 72 ± 1b | - | 74 ± 1cd | 74b | 75 ± 2cd | 74 ± 1bc | - | 73 ± 1c |
|  | 1-TZF5-72 | 75 ± 2b | 73b | 67 ± 0.3b | 72 ± 1d | 76 ± 2b | 72 ± 1d | 73c | 65 ± 1b | 75 ± 1bc |
|  | 14-1-2-10 BIL | - | 74 ± 1b | 68 ± 1a | 77 ± 1ab | - | 77 ± 1bc | 76 ± 1b | 66 ± 1ab | 79 ± 1a |
|  |  |  |  |  |  |  |  |  |  |  |
| Shoot biomass  (g m^-2^) | IR64 | 297 ± 27 | 629 ± 84a | 378 ± 17a | 429 ± 70ab | 241 ± 18c | 521 ± 25 ab | 565 ± 46a | 220 ± 18ab | 288 ± 9ab |
|  | 0-TZF5-13 | - | - | - | 311 ± 26bc | - | - | - | - | 230 ± 6b |
|  | 0-TZF5-24 | - | - | - | 363 ± 32abc | - | 548 ± 20 ab | - | - | 231 ± 20b |
|  | 0-TZF5-72 | - | - | - | 334 ± 64bc | - | - | - | - | 246 ± 20b |
|  | 1-TZF5-13 | 273 ± 67 | 500 ±39ab | - | 256 ± 51c | 301 ± 15bc | 441 ± 26 ab | 536 ± 26a | - | 290 ± 62ab |
|  | 1-TZF5-24 | 188 ± 24 | 379 ± 82b | - | 378 ± 60abc | 311 ± 20b | 571 ± 100 ab | 323 ± 130b | - | 232 ± 14b |
|  | 1-TZF5-72 | 224 ± 47 | 489 ± 74ab | 329 ± 13b | 429 ± 19ab | 391 ± 28a | 435 ± 59 b | 479 ± 22ab | 167 ± 24b | 220 ± 23b |
|  | 14-1-2-10 BIL | - | 666 ± 84a | 400 ± 10a | 508 ± 67a | - | 633 ± 88 a | 379 ± 47ab | 244 ± 8a | 358 ± 19a |
|  |  |  |  |  |  |  |  |  |  |  |
| Harvest index | IR64 | 0.41 ± 0.03 | 0.37 ± 0.04b | 0.53 ± 0.01ab | 0.50 ± 0.02 | 0.7 10-3c | 0.9 10^-3^b | 0.09 ± 0.03b | 0.09 ± 0.05 | 0.08 ± 0.03b |
|  | 0-TZF5-13 | **-** | **-** | **-** | 0.43 ± 0.06 | **-** | **-** | **-** | **-** | 0.26 ± 0.03a |
|  | 0-TZF5-24 | **-** | **-** | **-** | 0.48 ± 0.01 | **-** | 0.01 ± 0.01b | **-** | **-** | 0.09 ± 0.03b |
|  | 0-TZF5-72 | **-** | **-** | **-** | 0.45 ± 0.03 | **-** | **-** | **-** | **-** | 0.15 ± 0.03b |
|  | 1-TZF5-13 | 0.26 ± 0.06 | 0.41 ± 0.02b | **-** | 0.55 ± 0.08 | 0.18 ± 0.02a | 0.01 ± 0.01b | 0.26 ± 0.03ab | **-** | 0.06 ± 0.02b |
|  | 1-TZF5-24 | 0.37 ± 0.08 | 0.53 ± 0.04a | **-** | 0.46 ± 0.04 | 0.17 ± 0.02ab | 0.02 ± 0.01b | 0.28 ± 0.01a | **-** | 0.13 ± 0.04b |
|  | 1-TZF5-72 | 0.44 ± 0.11 | 0.45 ± 0.05ab | 0.51 ± 0.02b | 0.45 ± 0.03 | 0.09 ± 0.04b | 0.13 ± 0.04a | 0.24 ± 0.04ab | 0.23 ± 0.06 | 0.14 ± 0.04b |
|  | 14-1-2-10 BIL | **-** | 0.43 ± 0.03ab | 0.56 ± 0.01a | 0.50 ± 0.01 | **-** | 0.12 ± 0.04a | 0.36 ± 0.02a | 0.24 ± 0.03 | 0.07 ± 0.02b |
|  |  |  |  |  |  |  |  |  |  |  |

**Table S8.** Transgenic lines – Early generation trials (T_1_-T_3_): grain yield, time to flowering, shoot biomass and harvest index of IR64, nulls (0-TZF5), transgenic lines (1-TZF5) and 14-1-2-10 BIL in the screenhouse and field. das: days after sowing. Mean values ± se (*n* = 4) are presented and letters indicate significant difference groups. Sets with no letter groups indicate no significant differences among lines. Hyphen (-): Lines not included in the particular trial.

| **Traits** | **Line** | **Trials** |  |  |  |
| --- | --- | --- | --- | --- | --- |
|  |  | **SH4-W** | **SH5-W** | **SH4-VRS** | **SH5-SS** |
| Grain yield (g m^-2^) | IR64 | 441±96a | 348±32ab | 25±12c | 159±13abc |
|  | 0-TZF5-13 | 296±79a | **-** | 83±13ab | **-** |
|  | 0-TZF5-24 | 341±41a | **-** | 28±11c | **-** |
|  | 0-TZF5-72 | 320±79a | **-** | 53±14abc | **-** |
|  | 1-TZF5-13 | 308±41a | 151±29g | 21±6c | 88±9c |
|  | 1-TZF5-24 | 351±82a | 173±13efg | 38±13bc | 126±19bc |
|  | 1-TZF5-72 | 359±44a | 195±18defg | 41±15bc | 103±17c |
|  | 14-1-2-10 BIL | 511±62a | 391±25a | 29±6bc | 275±30a |
|  |  |  |  |  |  |
| Time to flowering (das) | IR64 | 78±0.7a | 73±1bcdef | 81±2a | 78±1abcd |
|  | 0-TZF5-13 | 72±0.5d | - | 72±2d | - |
|  | 0-TZF5-24 | 76±0.25ab | - | 77±1abc | - |
|  | 0-TZF5-72 | 75±1bc | - | 73±1cd | - |
|  | 1-TZF5-13 | 73±2cd | 82±2a | 78±3ab | 82±2ab |
|  | 1-TZF5-24 | 74±1cd | 78±1ab | 73±1cd | 79±1abc |
|  | 1-TZF5-72 | 72±0.6d | 70±1cdef | 75±0.9bcd | 79±3abc |
|  | 14-1-2-10 BIL | 77±0.5ab | 75±0.8abcd | 79±0.6a | 76±2abcd |
|  |  |  |  |  |  |
| Shoot biomass (g m^-2^) | IR64 | 428±69ab | 202±28abc | 298±9abc | 179±9a |
|  | 0-TZF5-13 | 320±30b | - | 258±9bc | - |
|  | 0-TZF5-24 | 360±31ab | - | 243±19bc | - |
|  | 0-TZF5-72 | 358±53ab | - | 285±11abc | - |
|  | 1-TZF5-13 | 263±36b | 91±10c | 298±49abc | 252±42a |
|  | 1-TZF5-24 | 379±58ab | 120±17bc | 241±13bc | 134±16a |
|  | 1-TZF5-72 | 421±20ab | 128±19bc | 233±20c | 170±48a |
|  | 14-1-2-10 BIL | 507±66a | 179±14bc | 371±16a | 205±14a |
|  |  |  |  |  |  |
| Harvest index | IR64 | 0.50±0.02 | 0.64±0.02abc | 0.07±0.03b | 0.47±0.02abc |
|  | 0-TZF5-13 | 0.46±0.05 | **-** | 0.24±0.03a | **-** |
|  | 0-TZF5-24 | 0.48±0.01 | **-** | 0.09±0.03b | **-** |
|  | 0-TZF5-72 | 0.46±0.03 | **-** | 0.15±0.03ab | **-** |
|  | 1-TZF5-13 | 0.54±0.07 | 0.60±0.07abcde | 0.07±0.02b | 0.28±0.06ac |
|  | 1-TZF5-24 | 0.46±0.04 | 0.59±0.02abcde | 0.13±0.04ab | 0.48±0.06abc |
|  | 1-TZF5-72 | 0.46±0.02 | 0.61±0.03abcde | 0.14±0.04ab | 0.40±0.07abc |
|  | 14-1-2-10 BIL | 0.50±0.01 | 0.69±0.01a | 0.07±0.02b | 0.57±0.02a |

**Table S9.** Transgenic lines – Later generation trials (T_4_-T_6_): grain yield, time to flowering, shoot biomass and harvest index of IR64, nulls (0-TZF5), transgenic lines (1-TZF5) and 14-1-2-10 BIL in the later-generation screenhouse trials. das: days after sowing. Mean±se are presented and letters indicate significant difference groups. The set with no letter groups indicates no significant differences among lines. Hyphen (-): Lines not included in the particular trial.

**Table S10.** Levels of polymorphisms in selected transgenic, pyramiding, and background-cleaned lines based on Infinium 6k or 7k (background-cleaned lines) SNP genotyping.

|  | Transgenic line | | Background-cleaned lines | | | | | | | Pyramided lines^$^ | | |
| --- | --- | --- | --- | --- | --- | --- | --- | --- | --- | --- | --- | --- |
| Chr # | 1-TZF5-72 vs IR64 | 1-TZF5-72 vs IR87707-445-B-B-B^$^ | IR64 x 1- TZF5-72 BC_3_F_1_ (-) | IR64 x 1- TZF5-24 BC_3_F_1_ (-) | IR64 x 1- TZF5-13 BC_3_F_1_ (-) | | IR64 x 1- TZF5-72 BC_3_F_1_ (+) | IR64 x 1- TZF5-24 BC_3_F_1_ (+) | IR64 x 1- TZF5-13 BC_3_F_1_ (+) | BC_2_F_4_-205-127 vs IR64^$^ | BC_2_F_4_-104-229 vs IR64^$^ | BC_2_F_4_-205-127 vs BC_2_F_4_-104-229^$^ |
|  | Levels of polymorphism (%) | | | | | | | | | | | |
| 1 | 10.1 | 5.1 | 0 | 0.76 | 0 | 0.06 | | 0.76 | 0.01 | 0.39 | 1.68 | 2.06 |
| 2 | 11.6 | 2.6 | 0 | 0.76 | 0 | 0.01 | | 0.75 | 0.00 | 1.13 | 0.81 | 0.97 |
| 3 | 4.9 | 4.7 | 0 | 0.75 | 0 | 0.01 | | 0.74 | 0.00 | 2.28 | 0.00 | 1.52 |
| 4 | 13.3 | 8.1 | 0 | 0.72 | 0 | 0.00 | | 0.70 | 0.02 | 3.50 | 2.27 | 2.27 |
| 5 | 4 | 2.6 | 0 | 0.75 | 0 | 0.00 | | 0.74 | 0.00 | 0.84 | 0.63 | 1.47 |
| 6 | 2.9 | 2.9 | 0 | 0.75 | 0 | 0.00 | | 0.73 | 0.07 | 3.55 | 4.73 | 8.28 |
| 7 | 4.1 | 8.7 | 0 | 0.81 | 0 | 0.00 | | 0.82 | 0.04 | 2.74 | 0.00 | 2.74 |
| 8 | 7.2 | 13.6 | 0 | 0.74 | 0 | 0.00 | | 0.73 | 0.00 | 1.01 | 5.63 | 4.43 |
| 9 | 0.3 | 3.3 | 0 | 0.76 | 0 | 0.00 | | 0.75 | 0.00 | 0.25 | 0.00 | 0.25 |
| 10 | 2.5 | 8.7 | 0 | 0.72 | 0 | 0.00 | | 0.71 | 0.00 | 0.00 | 0.71 | 0.71 |
| 11 | 5.2 | 6.4 | 0 | 0.74 | 0 | 0.00 | | 0.72 | 0.00 | 0.55 | 3.83 | 4.38 |
| 12 | 17.1 | 4.1 | 0 | 0.73 | 0 | 0.00 | | 0.70 | 0.00 | 0.00 | 0.00 | 0.00 |
| **Overall** | **6.9** | **5.9** | 0 | 0.75 | 0 | 0.01 | | 0.74 | 0.01 | 1.35 | 1.69 | 2.42 |

^$^qDTY2.2 and qDTY4.1 regions from Aday Sel are excluded

**Table S11.** Background cleaned lines - Days to flowering, shoot biomass, and plant height in one cylinder trial (C3) and one screenhouse trial (SH6). Each value represents the mean of three lines per genotype class and three (cylinders)/ four (screenhouse) replicates per line.

|  |  |  |  |  |  |
| --- | --- | --- | --- | --- | --- |
| **Traits** | **Genotype class** | **Trials** |  |  |  |
|  |  | C3-W | C3-VS | SH6-W | SH6-VS |
|  |  |  |  |  |  |
| Time to flowering (das) | IR64 x 1-TZF5-13 BC3F1 (-) | 56±0.8 c | - | 61±1 cd | 59±0.5.c |
|  | IR64 x 1-TZF5-24 BC3F1 (-) | 55±0.8 c | - | 61±1 cd | 57±0.5 d |
|  | IR64 x 1-TZF5-72 BC3F1 (-) | 55±0 c | - | 61±1 cd | 62±0.5 b |
|  | IR64 x 1-TZF5-13 BC3F1 (+) | 57±2 bc | - | 59±0 d | 57±0.5 cd |
|  | IR64 x 1-TZF5-24 BC3F1 (+) | 57±0.7 bc | - | 59±0 d | 56±0.5 d |
|  | IR64 x 1-TZF5-72 BC3F1 (+) | 59±2 abc | - | 63±1 bcd | 59±0.5 c |
|  | 0-TZF5-13 | 56±0.4 c | - | 68±2 ab | 61±0.5 b |
|  | 0-TZF5-24 | 57±0.5 bc | - | 66±0.3 abc | 62±0.5 b |
|  | 0-TZF5-72 | 58±0.8 abc | - | 69±2 a | 65±0.5 a |
|  | 1-TZF5-13 | 56±0.7 c | - | 66±0 abc | 62±0.5 b |
|  | 1-TZF5-24 | 56±0.7 c | - | 65±1 abcd | 62±0.5 b |
|  | 1-TZF5-72 | 60±0.3 ab | - | 68±1 ab | 65±0.5 a |
|  | IR64 | 61±1 a | - | 66±0.3 abc | 65±0.5 a |
|  |  |  |  |  |  |
|  |  |  |  |  |  |
| Shoot biomass (g m^2^) | IR64 x 1-TZF5-13 BC3F1 (-) | - | 10±0.3 ab | 301±27 a | 133±4 ab |
|  | IR64 x 1-TZF5-24 BC3F1 (-) | - | 11±0.8 a | 357±13 a | 172±14 ab |
|  | IR64 x 1-TZF5-72 BC3F1 (-) | - | 10±0.4 a | 419±18 a | 138±18 ab |
|  | IR64 x 1-TZF5-13 BC3F1 (+) | - | 10±0.5 a | 409±51 a | 231±106 ab |
|  | IR64 x 1-TZF5-24 BC3F1 (+) | - | 10±0.5 ab | 327±24 a | 178±9 ab |
|  | IR64 x 1-TZF5-72 BC3F1 (+) | - | 10±0.6 a | 292±16 a | 193±10 a |
|  | 0-TZF5-13 | - | 9±0.5 ab | 372±26 a | 204±46 a |
|  | 0-TZF5-24 | - | 8±0.5 b | 342±32 a | 172±10 ab |
|  | 0-TZF5-72 | - | 10±0.3 a | 516±134 a | 165±9 ab |
|  | 1-TZF5-13 | - | 10±0.5 ab | 344±24 a | 146±9 ab |
|  | 1-TZF5-24 | - | 12±3 ab | 373±22 a | 154±8 ab |
|  | 1-TZF5-72 | - | 10±0.5 ab | 549±198 a | 151±5 ab |
|  | IR64 | - | 10±0.5 ab | 267±7 a | 135±8 ab |
|  |  |  |  |  |  |
|  |  |  |  |  |  |
| Plant height (cm) | IR64 x 1-TZF5-13 BC3F2 (-) | - | 88±2 b | - | 95±1 b |
|  | IR64 x 1-TZF5-24 BC3F2 (-) | - | 92±3 ab | - | 98±2 b |
|  | IR64 x 1-TZF5-72 BC3F2 (-) | - | 91±3 b | - | 91±2 b |
|  | IR64 x 1-TZF5-13 BC3F2 (+) | - | 91±3 b | - | 96±1 b |
|  | IR64 x 1-TZF5-24 BC3F2 (+) | - | 87±2 b | - | 97±1 b |
|  | IR64 x 1-TZF5-72 BC3F2 (+) | - | 93±4 ab | - | 92±2 b |
|  | 0-TZF5-13 | - | 84±2 b | - | 93±1 b |
|  | 0-TZF5-24 | - | 84±1 b | - | 92±2 b |
|  | 0-TZF5-72 | - | 84±0.5 b | - | 100±1 b |
|  | 1-TZF5-13 | - | 85±3 b | - | 91±2 b |
|  | 1-TZF5-24 | - | 96±4 ab | - | 95±2 b |
|  | 1-TZF5-72 | - | 85±1 b | - | 98±2 b |
|  | IR64 | - | 89±2 b | - | 94±2 b |

**Table S12**. Pyramiding lines - Comparison of yield for each genotype across three experiments. Means±se are presented and letters indicate significant difference groups.

| Genotype | Combination of introgressions | SH7-RS | SH8-RS | SH9-RS | SH7-W | SH8-W | SH9-W |
| --- | --- | --- | --- | --- | --- | --- | --- |
| IR 125537-104-224 | + lipOsTZF5, +qDTY2.2,+qDTY4.1 | 377.4±45 a | 14.7±6 a | 233.0±35 ab | 315.9±39 ab | 534.2±66 b | 662.9±49 bcd |
| IR 125537-104-229 | + lipOsTZF5, +qDTY2.2,+qDTY4.1 | 350.7±38 ab | 60.1±9 a | 232.2±22 ab | 326.7±12 ab | 380.6±81 cd | 687.3±33 bcd |
| IR 125537-104-308 | + lipOsTZF5, +qDTY2.2, qDTY4.1 | 298.8±45 abcd | 53.7±12 a | 196.6±39 bcd | 298.2±24 ab | 544.5±85 b | 609.4±36 cd |
| IR 125537-104-324 | + lipOsTZF5, +qDTY2.2,+qDTY4.1 | 295.9±10 abcd | 28.6±12 a | 185.8±34 bcd | 294.4±42 ab | 224.5±10 e | 583.8±42 d |
| IR 125537-104-348 | + lipOsTZF5, +qDTY2.2,+qDTY4.1 | 295.5±31 abcd | 55.5±12a | 242.5±24 ab | 278.6±35 ab | 367.0±92 d | 763.±65 ab |
| IR 125537-160-51 | + lipOsTZF5, +qDTY2.2,+qDTY4.1 | 216.5±44 d | 54.0±27a | 294.4±30 a |  | 564.0±101 b | 685.1±40 bcd |
| IR 125537-205-127 | + lipOsTZF5, +qDTY2.2, qDTY4.1 | 261.3±38 cd | 91.9±22 a | 197.0±22 bcd | 358.9±39 a | 500.4±51 bc | 590.1±33 d |
| IR 125537-104-56 | + lipOsTZF5, +qDTY2.2 | 341.4±6 abc | 64.9±2 a | 226.8±19 abc | 388.4±9 a | 381.6±57 cd | 612.6±51 cd |
| IR 125537-104-22 | + lipOsTZF5, +qDTY4.1 | 368.2±55 ab | 62.9±2 a | 210.6±41 bcd |  | 513.7±57 b | 814.1±32 a |
| IR 125537-104-219 | - lip9:OsTZF5 (null) | 318.8±30 abc | 61.4±15 a | 207.6±22 bcd | 319.0±63 ab | 497.3±70 bc | 730.3±53 abc |
| 1-TZF5-72 | transgenic line | 124.7±17 e | 11.5±5 a | 146.7±18 cd | 149.7±69 cd | 464.6±38 bcd | 574.3±7 d |
| IR64 | wild type | 286.5±17 bcd | 66.4±24 a | 214.9±55 abcd | 203.7±82 bc | 491.4±39 bc | 616.6±25 cd |
| IR87707-445-B-B-B | QTL NIL | 326.1±37 abc | 59.3±47 a | 134.1±52 d | 375.6±17 a | 764.7±59 a | 825.9±43 a |
|  | | | |  |  |  |  |

**Table S13.** Pyramiding lines  - Comparison of days to flowering and shoot biomass for each genotype across three experiments. Means are presented and letters indicate significant difference groups.

|  |  | | | | | | |  |  |
| --- | --- | --- | --- | --- | --- | --- | --- | --- | --- |
| Genotype | Combination of introgressions | SH7-RS | SH8-RS | SH9-RS | SH7-W | SH8-W | SH9-W |  |  |
| Days to flowering |  |  |  |  |  |  |  |  |  |
| IR 125537-104-224 | + lipOsTZF5, +qDTY2.2,+qDTY4.1 | 55.7cd | 61.7bc | 53.7cd | 58.3b | 64.3cd | 54.7e |  |  |
| IR 125537-104-229 | + lipOsTZF5, +qDTY2.2,+qDTY4.1 | 54.7d | 60.7cd | 53.7cd | 58.7b | 64.0cde | 55.3de |  |  |
| IR 125537-104-308 | + lipOsTZF5, +qDTY2.2,+qDTY4.1 | 54.7d | 60.3cd | 53.5cd | 57.7b | 62.0ef | 56.3cde |  |  |
| IR 125537-104-324 | + lipOsTZF5, +qDTY2.2, qDTY4.1 | 55.0cd | 61.0bcd | 54.3bc | 58.3b | 64.7bc | 56.3cde |  |  |
| IR 125537-104-348 | + lipOsTZF5, +qDTY2.2,+qDTY4.1 | 54.7d | 60.0d | 52.7d | 58.3b | 62.0ef | 56.3cde |  |  |
| IR 125537-160-51 | + lipOsTZF5, +qDTY2.2,+qDTY4.1 | 56.0c | 62.3b | 52.7d | 58.7b | 62.7cdef | 55.0de |  |  |
| IR 125537-104-56 | + lipOsTZF5, +qDTY2.2 | 55.0cd | 61.0bcd | 52.7d | 58.0b | 66.7b | 57.0cd |  |  |
| IR 125537-205-127 | + lipOsTZF5, +qDTY2.2, qDTY4.1 | 55.7cd | 60.0d | 52.7d | 57.7b | 60.7f | 55.7de |  |  |
| IR 125537-104-22 | + lipOsTZF5, +qDTY4.1 | 55.0cd | 60.3cd | 52.7d | 58.3b | 62.3def | 56.3cde |  |  |
| IR 125537-104-219 | - lip9:OsTZF5 (null) | 54.7d | 60.0d | 52.7d | 57.7b | 63.3cde | 55.3de |  |  |
| 1-TZF5-72 | transgenic line | 58.3b | 62.3b | 55.3b | 63.2a | 66.7b | 60.3b |  |  |
| IR64 | wild type | 55.7cd | 61.3bcd | 55.7b | 59.0b | 64.3cd | 58.3bc |  |  |
| IR87707-445-B-B-B | QTL NIL | 61.7a | 66.7a | 61.7a | 63.3a | 69.3a | 64.3a |  |  |
|  |  |  |  |  |  |  |  |  |  |
| Shoot biomass (g m^-2^) |  |  |  |  |  |  |  |  |  |
| IR 125537-104-224 | + lipOsTZF5, +qDTY2.2,+qDTY4.1 | 345.3bc | 596.4bc | 287.3a | 410.4abc | 587.4a | 633.5a |  |  |
| IR 125537-104-229 | + lipOsTZF5, +qDTY2.2,+qDTY4.1 | 303.2cd | 704.9ab | 331.7a | 391.0abc | 861.1a | 633.7a |  |  |
| IR 125537-104-308 | + lipOsTZF5, +qDTY2.2,+qDTY4.1 | 381.3ab | 610.6abc | 349.6a | 358.2bc | 544.5a | 561.5a |  |  |
| IR 125537-104-324 | + lipOsTZF5, +qDTY2.2, qDTY4.1 | 436.9a | 531.6bc | 326.6a | 425.8abc | 464.9a | 603.5a |  |  |
| IR 125537-104-348 | + lipOsTZF5, +qDTY2.2,+qDTY4.1 | 301.1cd | 479.3bc | 384.2a | 309.9c | 433.7a | 468.1a |  |  |
| IR 125537-160-51 | + lipOsTZF5, +qDTY2.2,+qDTY4.1 | 334.0bc | 565.3bc | 521.2a | 380.8abc | 443.1a | 538.4a |  |  |
| IR 125537-104-56 | + lipOsTZF5, +qDTY2.2 | 331.3bc | 548.7bc | 314.9a | 386.8abc | 426.5a | 510.7a |  |  |
| IR 125537-205-127 | + lipOsTZF5, +qDTY2.2, qDTY4.1 | 283.5cd | 452.8c | 366.8a | 425.6abc | 671.8a | 591.3a |  |  |
| IR 125537-104-22 | + lipOsTZF5, +qDTY4.1 | 350.2bc | 678.1abc | 304.4a | 347.1bc | 526.6a | 670.3a |  |  |
| IR 125537-104-219 | - lip9:OsTZF5 (null) | 295.1cd | 438.0c | 319.7a | 357.3bc | 508.4a | 569.2a |  |  |
| 1-TZF5-72 | transgenic line | 379.6ab | 561.0bc | 376.6a | 458.0ab | 550.9a | 475.6a |  |  |
| IR64 | wild type | 248.0d | 451.2c | 366.9a | 309.1c | 557.8a | 484.5a |  |  |
| IR87707-445-B-B-B | QTL NIL | 423.3a | 840.1a | 387.8a | 505.2a | 679.4a | 693.0a |  |  |

*^#^* Means with the same letter are not significant.

**Table S14.** Transgenic lines - Transpiration related traits in IR64 and 1-TZF5 transgenic lines during the field and screenhouse trials. Increase of canopy temperature was calculated as the slope of canopy temperature over time after initiation of the stress in the drought treatment. Stomatal conductance was measured at 69, 75, 68, 91 and 64 days after sowing in Trials SH1-W, SH1-S, F-W, F-VRS, SH2-W and SH2-S, respectively. Maximum quantum efficiency of photosystem II was measured at 70, 75, 81 and 70 days after sowing in Trials FW, F-VRS, F-RS and SH2-S, respectively. Relative water content and leaf water potential was measured at the same time at 76 and 81 days after sowing in trials F-VRS, SH2-W and SH2-S, respectively. Mean values ± se are presented. Significant differences among lines were only observed for stomatal conductance in trial F-VRS; no significant differences among lines were observed for all other measurements shown.

| **Lines** |  | **Increase in canopy temperature** | | | | | | |
| --- | --- | --- | --- | --- | --- | --- | --- | --- |
|  | **Trial** | SH1-S |  | F-VRS |  | F-RS |  | SH2-S |
| 1-TZF5-13 |  | 0.28 ± 0.05 |  | 0.16 ± 0.08 |  | 0.43 ± 0.09 |  | - |
| 1-TZF5-24 |  | 0.30 ± 0.04 |  | 0.23 ± 0.08 |  | 0.51 ± 0.13 |  | - |
| 1-TZF5-72 |  | 0.30 ± 0.03 |  | 0.31 ± 0.07 |  | 0.45 ± 0.15 |  | 0.28 ± 0.02 |
| IR64 |  | 0.32 ± 0.05 |  | 0.11 ± 0.08 |  | 0.47 ± 0.09 |  | 0.27 ± 0.05 |

| **Lines** |  | **Stomatal conductance (mmol m^-2^ s^-1^)** | | | | | | | | | | |
| --- | --- | --- | --- | --- | --- | --- | --- | --- | --- | --- | --- | --- |
|  | **Trial** | SH1-W |  | SH1-S |  | F-W |  | F-VRS |  | SH2-W |  | SH2-S |
| 1-TZF5-13 |  | 715 ± 56 |  | 229 ± 6 |  | 725 ± 5 |  | 66 ± 15b |  | - |  | - |
| 1-TZF5-24 |  | 770 ± 33 |  | 263 ± 7 |  | 730 ± 6 |  | 97 ± 9ab |  | - |  | - |
| 1-TZF5-72 |  | 775 ± 22 |  | 229 ± 12 |  | 706 ± 20 |  | 174 ± 47a |  | 622 ± 8 |  | 60 ± 30 |
| IR64 |  | 735 ± 43 |  | 258 ± 31 |  | 715 ± 15 |  | 140 ± 12ab |  | 641 ± 13 |  | 21 ± 10 |

| **Lines** |  | **Maximum quantum efficiency of Photosystem II (Fv/Fm)** | | | | | | |
| --- | --- | --- | --- | --- | --- | --- | --- | --- |
|  | **Trial** | F-W |  | F-VRS |  | F-RS |  | SH2-S |
| 1-TZF5-13 |  | 0.63 ± 0.08 |  | 0.70 ± 0.07 |  | 0.62 ± 0.07 |  | - |
| 1-TZF5-24 |  | 0.63 ± 0.05 |  | 0.68 ± 0.07 |  | 0.67 ± 0.04 |  | - |
| 1-TZF5-72 |  | 0.61 ± 0.06 |  | 0.74 ± 0.05 |  | 0.54 ± 0.05 |  | 0.31 ± 0.02 |
| IR64 |  | 0.64 ± 0.02 |  | 0.67 ± 0.04 |  | 0.54 ± 0.09 |  | 0.32 ± 0.02 |

| **Lines** |  | **Relative water content (%)** | | |  | **Leaf water potential (-MPa)** | | | | |
| --- | --- | --- | --- | --- | --- | --- | --- | --- | --- | --- |
|  | **Trial** | F-VRS |  | SH2-S |  | F-VRS |  | SH2-W |  | SH2-S |
| 1-TZF5-13 |  | 79.6 ± 5.1 |  | - |  | 1.72 ± 0.28 |  | - |  | - |
| 1-TZF5-24 |  | 79.8 ± 6.7 |  | - |  | 1.81 ± 0.09 |  | - |  | - |
| 1-TZF5-72 |  | 76.7 ± 14 |  | 11.26 ± 1.2 |  | 1.77 ±0.87 |  | 2.84 ± 0.09 |  | 3.41 ± 0.59 |
| IR64 |  | 66.4 |  | 9.10 ± 2.5 |  | 1.92 ± 0.12 |  | 1.98 ± 0.29 |  | 4.0 |

A

**
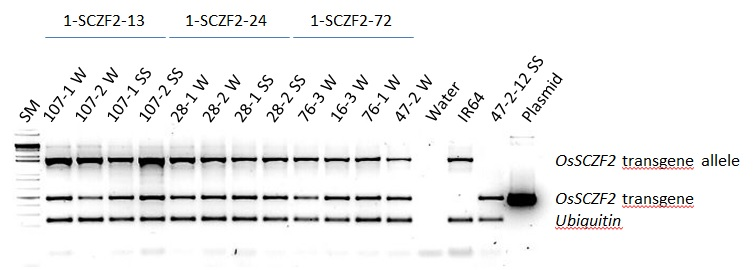
**

**
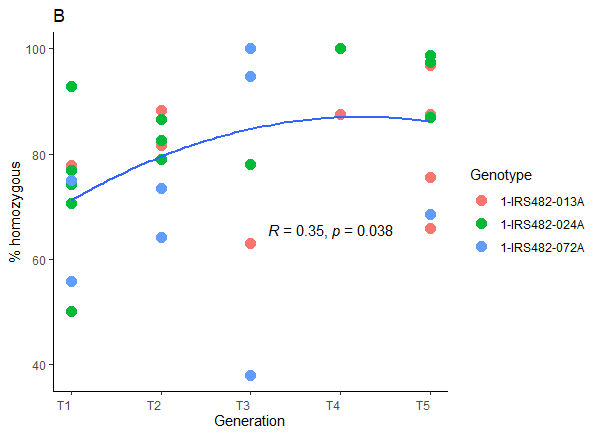
**

**Figure S1.** PCR amplification of the hygromycin gene or *OsTZF5* transgene was used to identify transgene positive plants, and to quantify the percentage of homozygous plants in each trial. A) Heterozygous *OsTZF5* transgenic T2 seeds of 1-TZF5-13, 1-TZF5-24 and 1-TZF5-72 in Trial SH1. Seed DNA from individual T1 transgenic plants growing under well-watered conditions (W) or seedling stage drought stress (SS) were used for multiplex PCR using primers detecting the *OsTZF5* transgene allele, *OsTZF5* transgene, and *Ubiquitin* gene as internal control. Numbers indicate plot and hill identity in Trial SH1. 47-2-12 SS is a control T4 homozygous line. SM: 1Kb plus DNA ladder. B) Homozyosity percentages over the generations grown in this study based on the proportion of seedlings for which the hygromycin gene was positive. Each symbol represents the homozygosity level of one transgenic event in one trial.

**
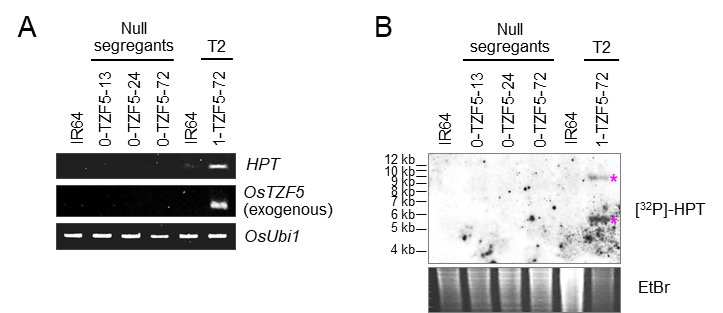
**

**Figure S2.** Validation of the absence of T-DNA insertions in azygous null segregant lines.

(A) Genomic PCR analysis of *HPT* gene and exogenous *OsTZF5* gene in the null segregant lines, 0-TZF5-13, 0-TZF5-24, and 0-TZF5-72. Wild-type IR64 and the T2 homozygous line of 1-TZF5-72 were used as negative or positive controls. *OsUbi1* was amplified as an internal control.

(B) Southern blot analysis of null segregant lines. *Eco*RI-digested genomic DNAs were hybridized with the [^32^P]-labeled HPT probe for Southern blot analysis. The ethidium bromide-stained gel was imaged as a loading control before membrane hybridization. Specific bands for 1-TZF5-72 are marked by asterisks. Positions according to the size marker are shown.


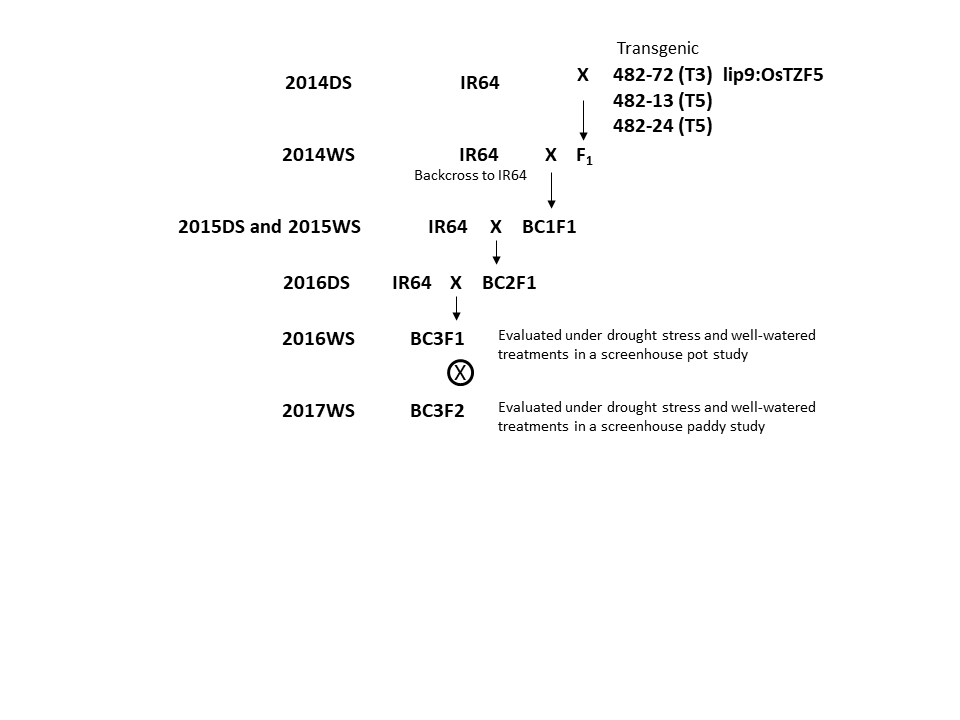


**Figure S3.** Overall summary of the background cleaning of events *LIP9:OsTZF5-13, LIP9:OsTZF5-24,* and *LIP9:OsTZF5-72* by backcrossing to IR64.


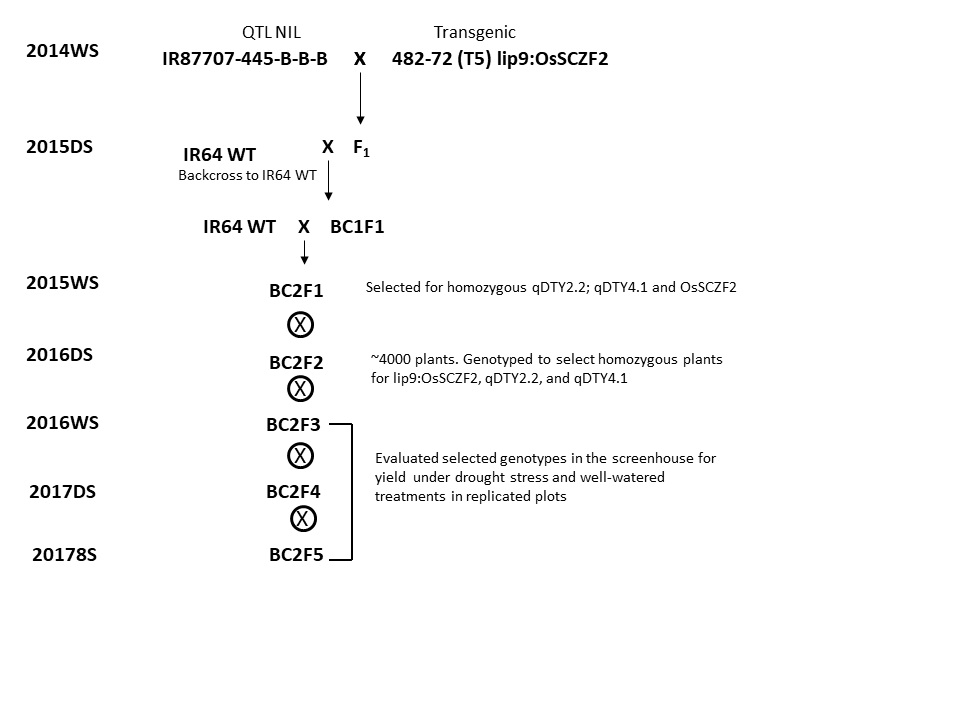


**Figure S4.** Overall summary of crossing work in the qDTY2.2 and 4.1 NIL IR87707-445-B-B-B × lead transgenic event 1-TZF5-72 crossing scheme.

**Figure S5.** Flanking sequence and Southern blot analysis of 1-TZF5 transgenic lines. (A) Description of the T-DNA arrangement models predicted by TAIL-PCR analysis in 1-TZF5-13, 1-TZF5-24 and 1-TZF5-72. 1-TZF5-13 contains two inverted T-DNA tandem repeats inserted within 6.2 kb interval on chromosome 10 (BAC clone OSJNBb0048O22 with NCBI accession number AC099325). Allele 1 was located in the coding sequence of LOC_Os10g13700 coding for a putative phosphoenolpyruvate carboxykinase or in the 3' untranslated region (3'UTR) of LOC_Os10g13694 coding for an unknown protein. Allele 2 was located in an intergenic region between LOC_Os10g13700 and OSJNBb0048O22.18, each encoding a hypothetical protein. Southern blot analysis suggested that allele 1 was partial, but genotyping confirmed the presence of the two full-length copies of the LIP9:OsTZF5 transgene at this allele. In 1-TZF5-24, one T-DNA was inserted on chromosome 7 (PAC clone P0006G05 with NCBI accession number AP004235) in the 3'UTR of LOC_Os07g36170 coding a putative chitin-inducible gibberellin-responsive protein, 1.6 kb upstream of LOC_Os07g36180 coding a putative pentatricopeptide. In 1-TZF5-72, one inverted T-DNA tandem repeat was identified on chromosome 3 (BAC clone OJ1175C11 with NCBI accession number AC10389) in an intergenic region between LOC_Os03g13040 coding a putative haemolysin-III related protein and LOC_Os03g13050 coding a putative E2F-related protein. (B) The insertion model based on TAIL-PCR was confirmed by Southern blot. Digestion patterns of the genomic DNA by *Eco*RI and *Bam*HI, and band size predicted for the Southern blot analysis using the probe for HPT region are shown. *Eco*RI, *Bam*HI, or *Eco*RI and *Bam*HI-digested genomic DNAs of IR64 and 1-TZF5-13, 1-TZF5-24, and 1-TZF5-72 lines were hybridized with the HPT probe for region Southern blot analysis. Positions according to the size marker are shown. (C) Genomic PCR for the allele 1 of 1-TZF5-13 using primer sets described in (A). Specific bands for 1-TZF5-13 are marked by asterisks.

**
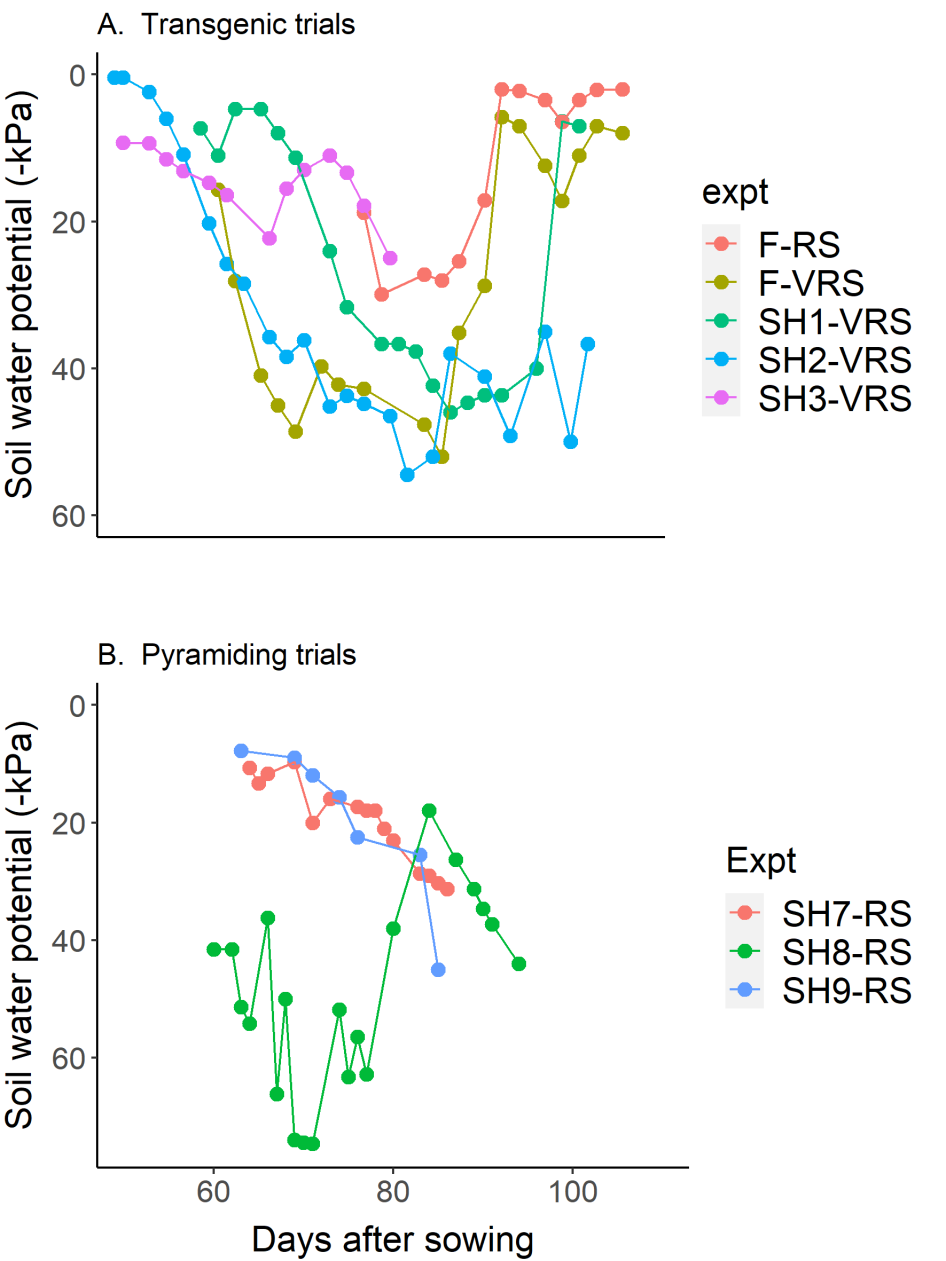
**

**Figure S6.** Soil water potential readings in A) transgenic trials, and B) the pyramiding trial. Tensiometers were installed at a depth of 30 cm. Mean values of *n* = 4 (Trials SH-1-VRS, SH2-VRS and SH-3-VRS) and *n* = 11 (Trials F-VRS and F-RS) tensiometer readings are presented. Arrows indicate rewatering dates for Trials SH1-VRS, F-VRS and F-RS. Trials SH2-VRS and SH3-VRS were not rewatered. The drought stress in the background cleaned trial (SH6-RS) was mild with a minimum soil water potential of -11 kPa at the 30 cm depth.

SH: screenhouse, F: field

RS: reproductive stage drought stress, VRS: vegetative and reproductive stage drought stress

**
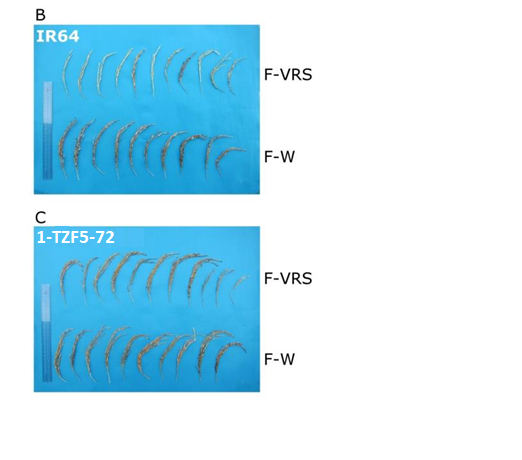
**

**A**

**B**

**Figure S7.** Representative pictures of the agronomic performance of 1-TZF5-72 in the field trial. Pictures show the total number of panicles from one IR64 (A) and 1-TZF5-72 (B) plant in the drought stress (F-VRS) and well-watered (F-W) treatments.


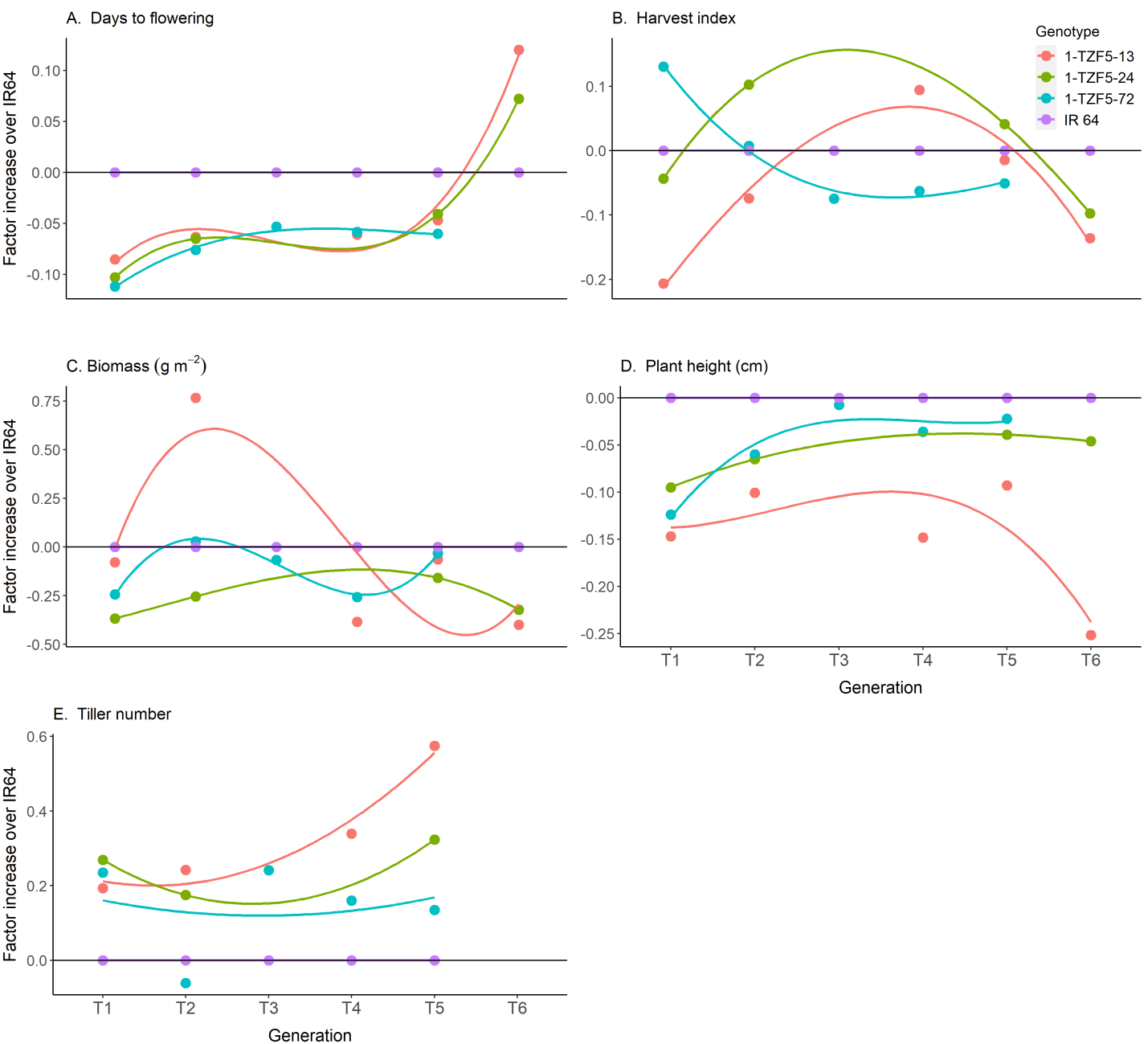


**Figure S8.** Generational trends of the transgenics in the well-watered treatments: A) days to flowering, B) harvest index, C) straw biomass at harvest, D) plant height, and E) tiller number. Mean values per generation are shown which overlapped across experiments and thus were not compared statistically.

**
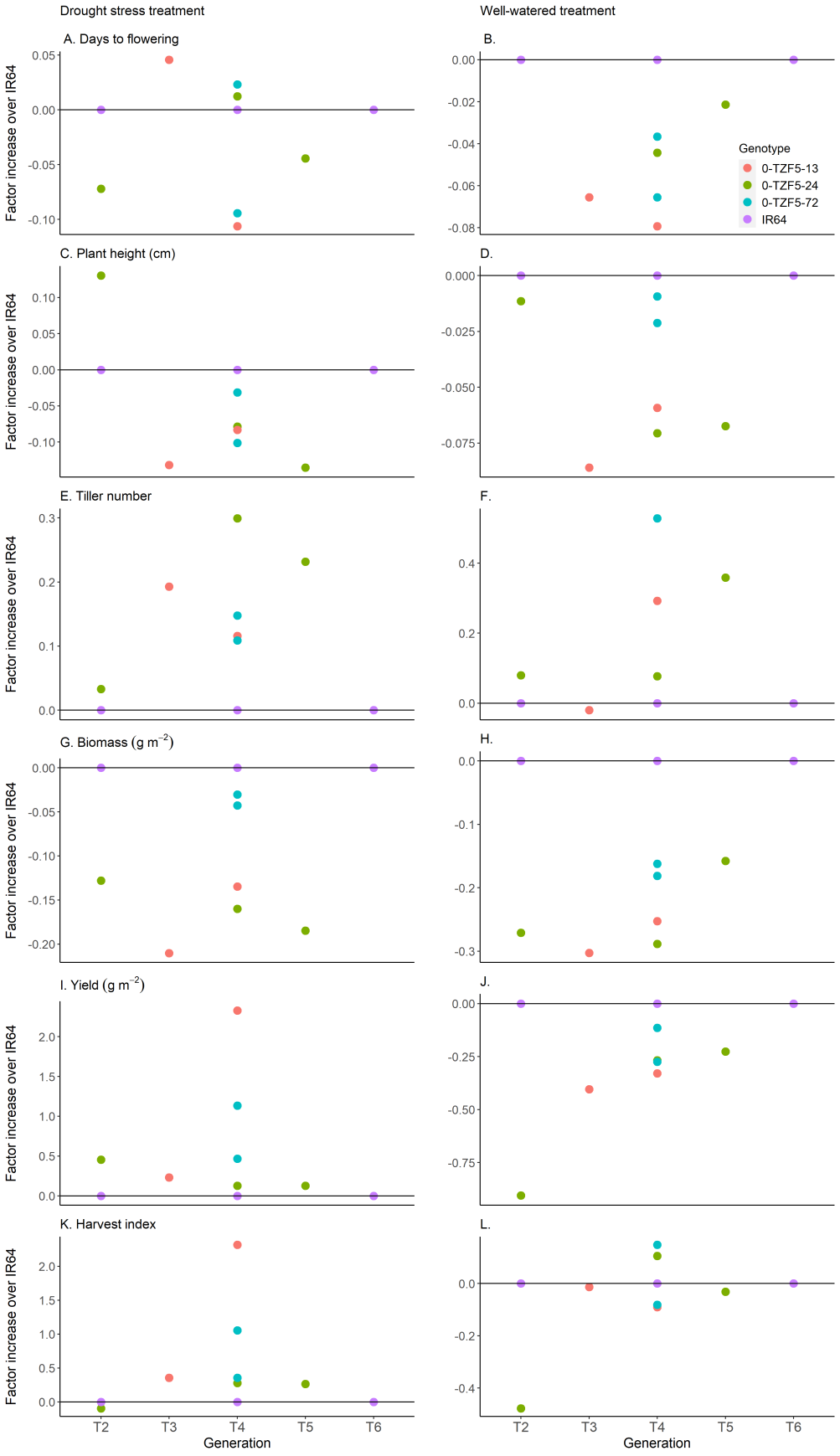
**

**Figure S9.** Null lines - Generational trends in agro-morphological traits across screenhouse and field trials in the drought stress (left) and well-watered (right) treatments. Each value represents the mean of factor increase over IR64. Corresponding data of the transgenic lines and IR64 are shown in Figs 2 and S8.

**
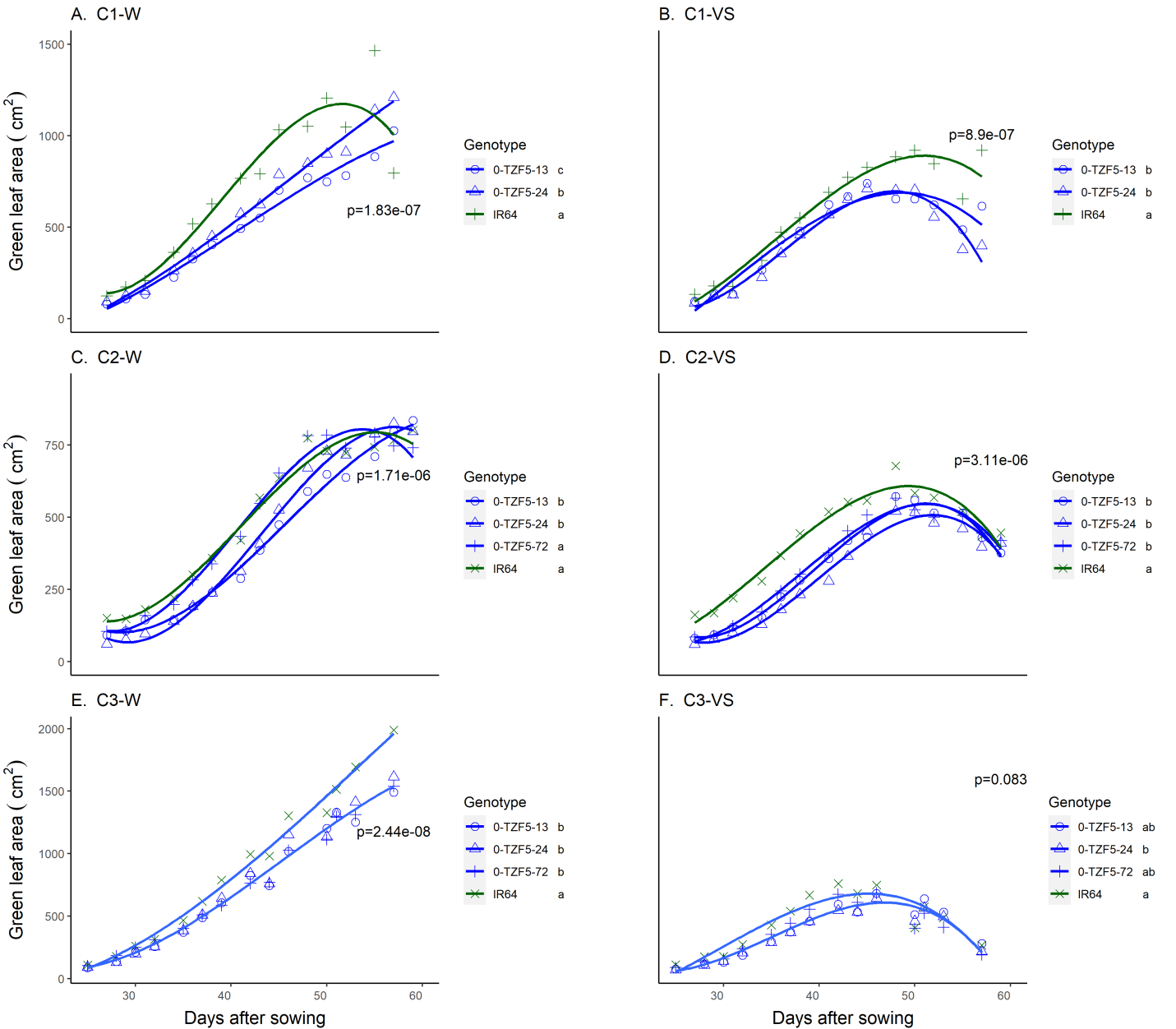
**

**Figure S10.** Null lines - Green leaf area across cylinder studies in null lines. Each value represents the mean of four replicates per genotype, and letters indicate significance groups based on a comparison of null and IR64. Corresponding data of the transgenic lines and IR64 are shown in Fig. 4.


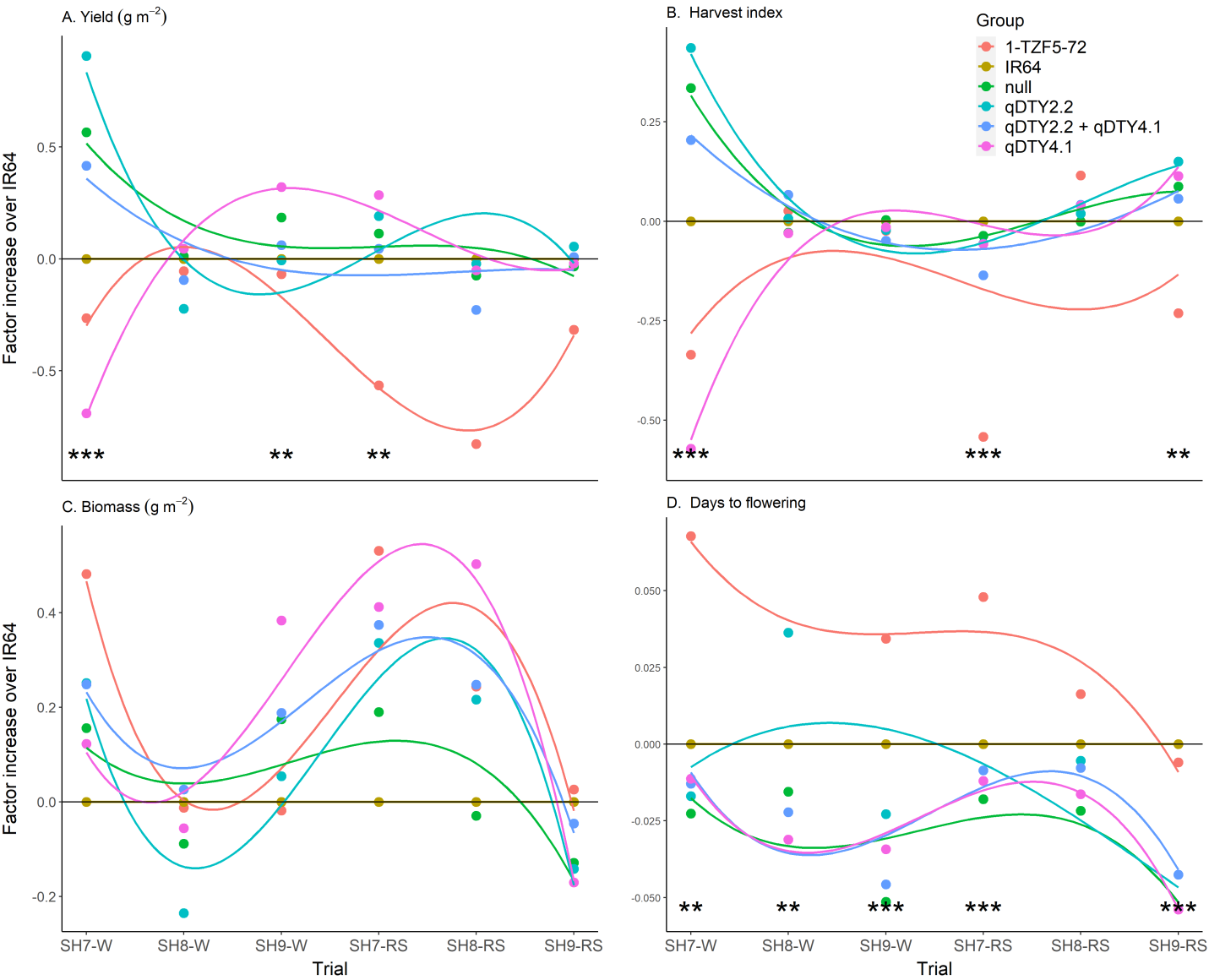


**Figure S11.** Pyramided lines factor increase over IR64 for agronomic traits: A) grain yield, B) harvest index, C) straw biomass at harvest, and D) days to flowering. The generations of the transgenic line 1-OsTZF5-072A shown are from BC_2_F_3_ – BC_2_F_5_. All lines shown were positive for the *OsTZF5* transgene except ‘IR64’ and ‘null’. Symbols represent mean values per line/QTL group, and the values were compared by ANOVA for each experiment and treatment. Differences among lines/QTL groups are indicated by * (*p* < 0.05), ** (*p* < 0.01), and *** (*p* < 0.001).

SH: screenhouse, F: field

W: well-watered, RS: reproductive stage drought stress

IR64: drought susceptible wild-type, 1- T-TZF5-72: transgenic line, qDTY2.2: pyramided line with qDTY2.2 and OsTZF5, qDTY2.2 + qDTY4.1: pyramided line with qDTY2.2, qDTY4.1 and OsTZF5, qDTY4.1: pyramided line with qDTY4.1 and OsTZF5

**
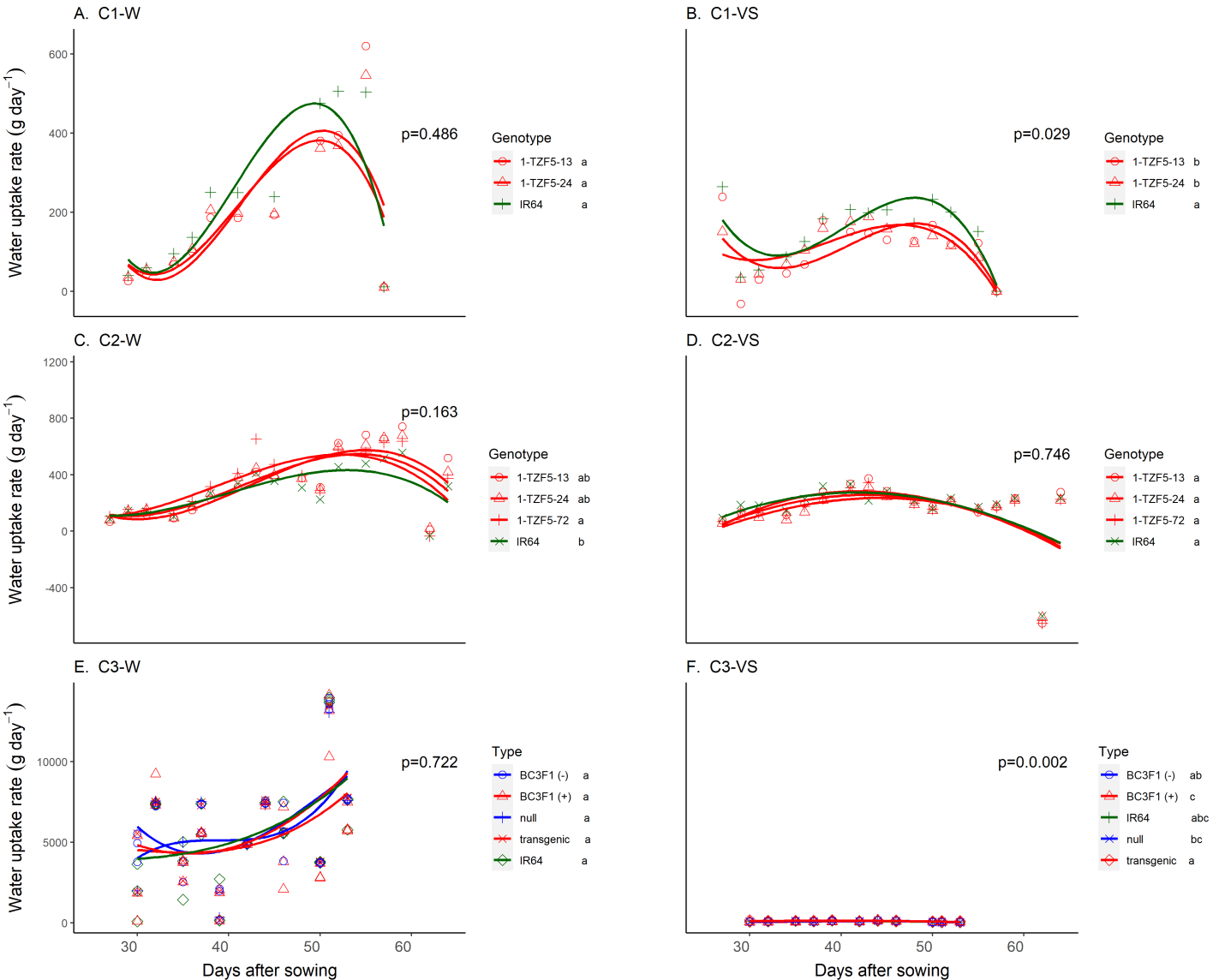
**

**Figure S12.** Water uptake rates in the cylinder studies of the transgenic lines (A-B: Trial C1 and C-D: Trial C2) and background-cleaned lines (E-F: Trial C3).Water uptake rates were calculated as the amount of water loss from the cylinder divided by the number of days between two successive measurements. Mean values (*n* = 4) are presented. Symbols represent means per transgenic line or genotype group and were compared across dates by ANOVA. Significant differences among lines/genotype groups are indicated by the letters next to the legend in each panel.

C: Cylinder

W: well-watered, VS: vegetative stage drought stress
IR64: drought susceptible wild-type,  1-TZF5-13, 1-TZF5-24, 1- T-TZF5-72: transgenic lines, BC3F1(-): background-cleaned lines without the transgene, BC3F1(+): background-cleaned lines with the transgene, transgenic: 1-TZF5-13, 1-TZF5-24, and 1- T-TZF5-72


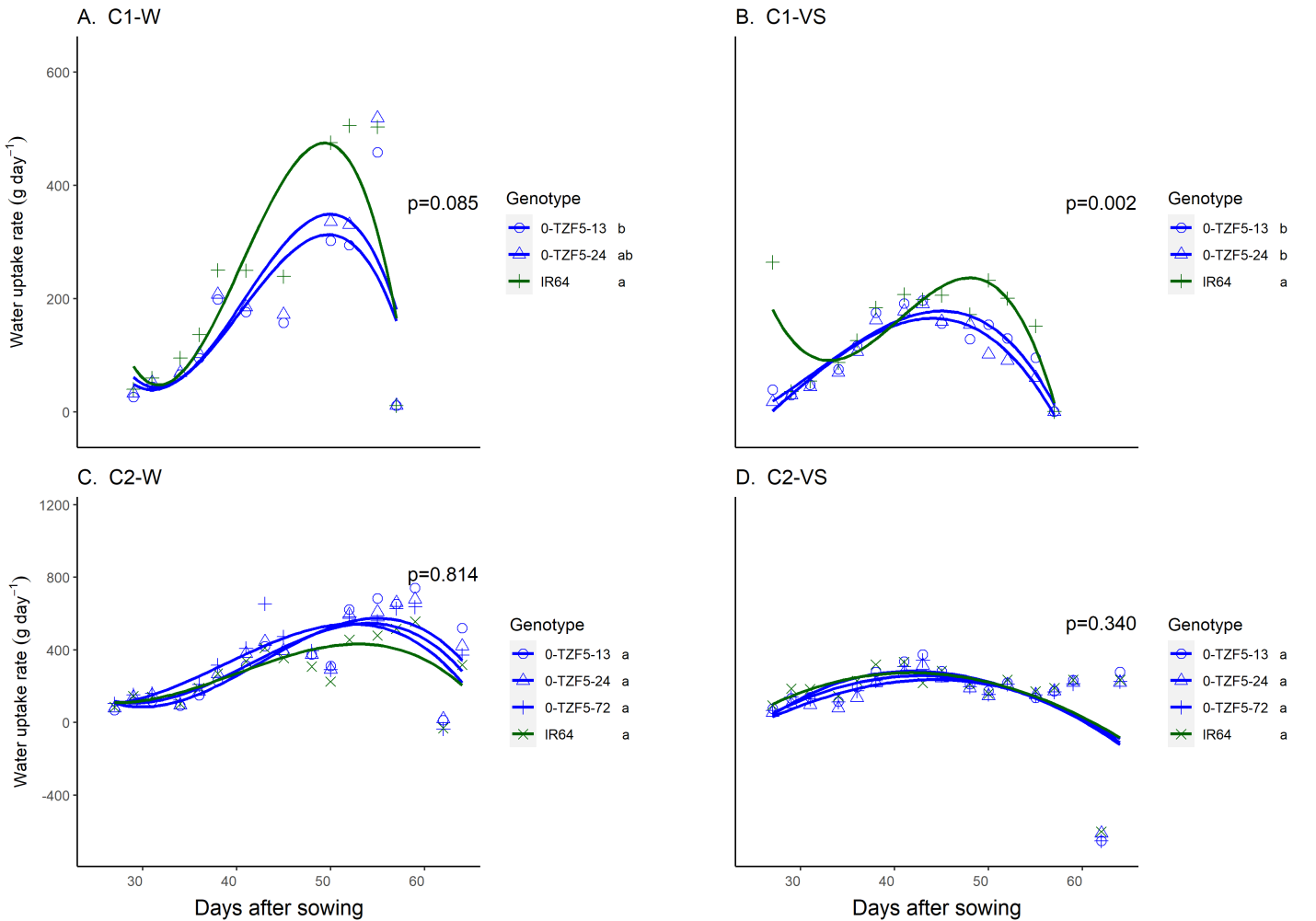


**Figure S13.** Water uptake rates in the cylinder studies of the null lines (A-B: Trial C1 and C-D: Trial C2).Water uptake rates were calculated as the amount of water loss from the cylinder divided by the number of days between two successive measurements. Mean values (*n* = 4) are presented. Symbols represent means per null line and IR64 were compared across dates by ANOVA. Significant differences among lines/genotype groups are indicated by the letters next to the legend in each panel.

C: Cylinder

W: well-watered, VS: vegetative stage drought stress
IR64: drought susceptible wild-type, 1-TZF5-13, 1-TZF5-24, 1- T-TZF5-72: transgenic lines, BC3F1(-): background-cleaned lines without the transgene, BC3F1(+): background-cleaned lines with the transgene, transgenic: 1-TZF5-13, 1-TZF5-24, and 1- T-TZF5-72

**
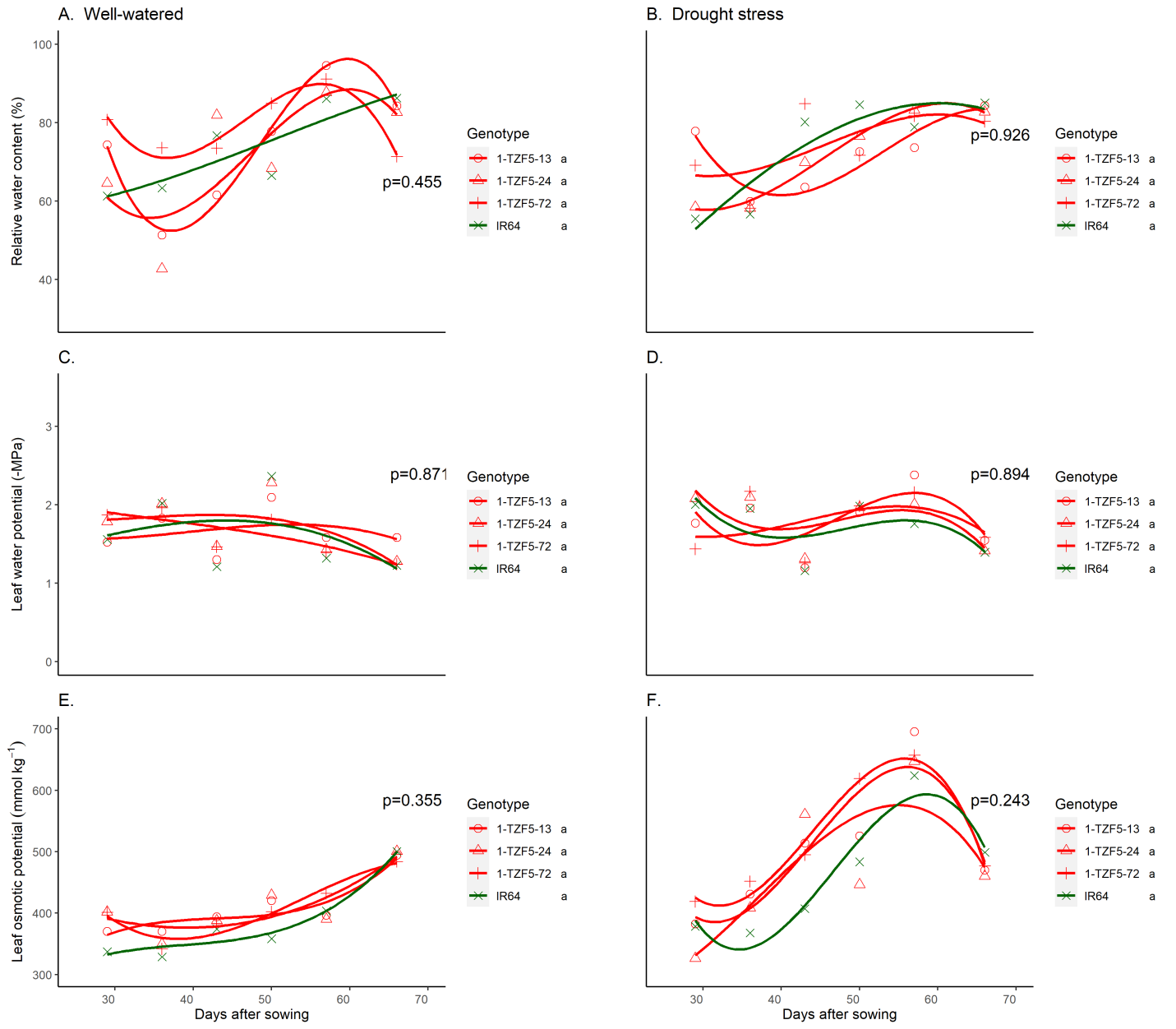
**

**Figure S14.** Transgenic lines - Leaf water status. Relative water content, leaf water potential, and leaf osmotic potential of IR64, and transgenic lines (1-TZF5-13, 1-TZF5-24 and 1-TZF5-72) in cylinders. Measurement were performed during Trial C2 at 29, 36, 43, 50, 57 and 66 das on fully-expanded leaves of plants grown under well-watered (A, C and E) and drought stress (B, D and F) conditions. Symbols represent means (*n* = 4) per transgenic line and were compared across dates by ANOVA. Significant differences among lines are indicated by the letters next to the legend in each panel.

IR64: drought susceptible wild-type,  1-TZF5-13, 1-TZF5-24, 1- T-TZF5-72: transgenic lines

**
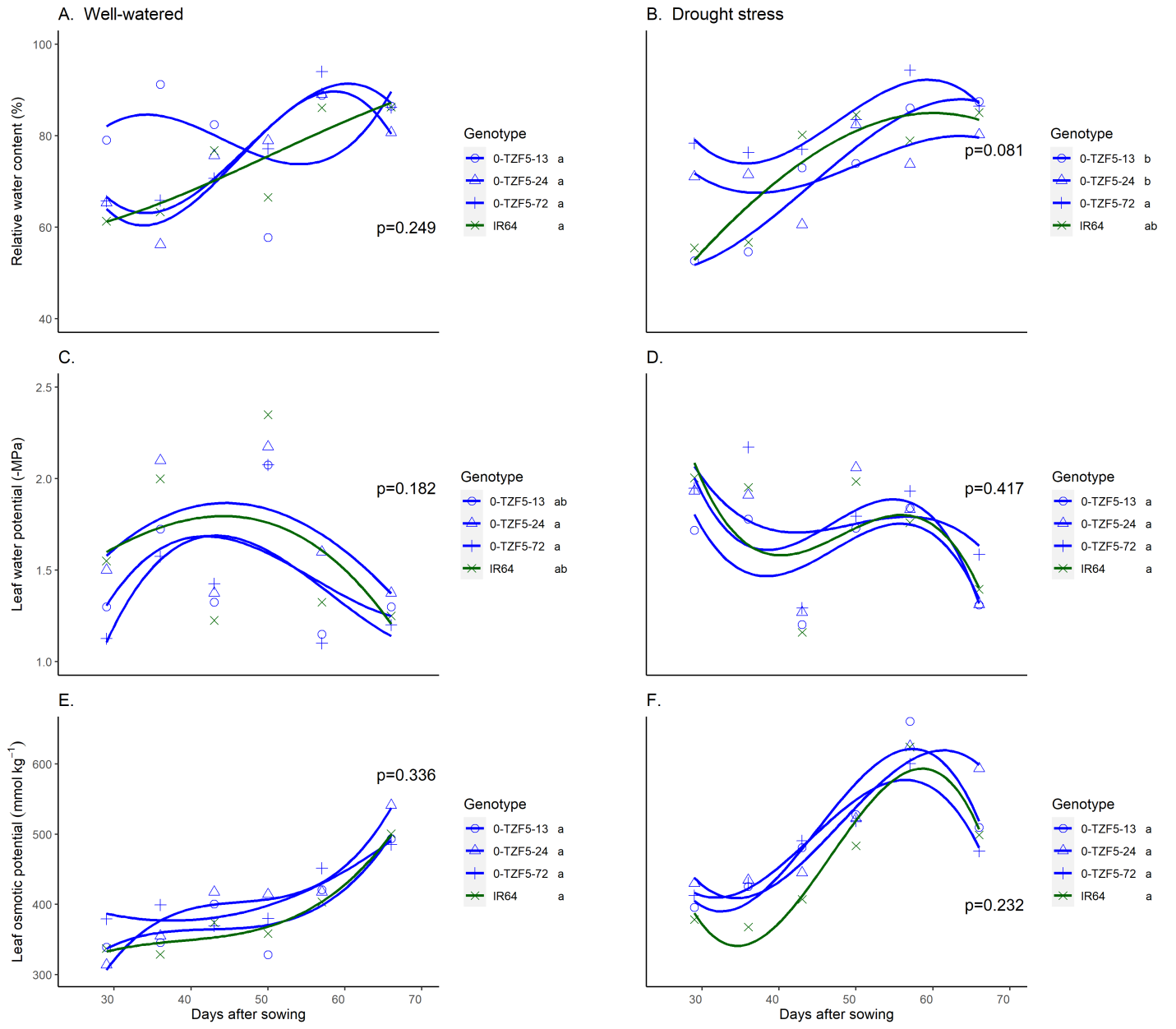
**

**Figure S15.** Null lines - Leaf water status. Relative water content, leaf water potential, and leaf osmotic potential of null lines in cylinder C2 trial. Letters indicate significance groups based on a comparison of the null lines and IR64 across measurement dates. Corresponding data of the transgenic lines and IR64 are shown in Fig. S14.


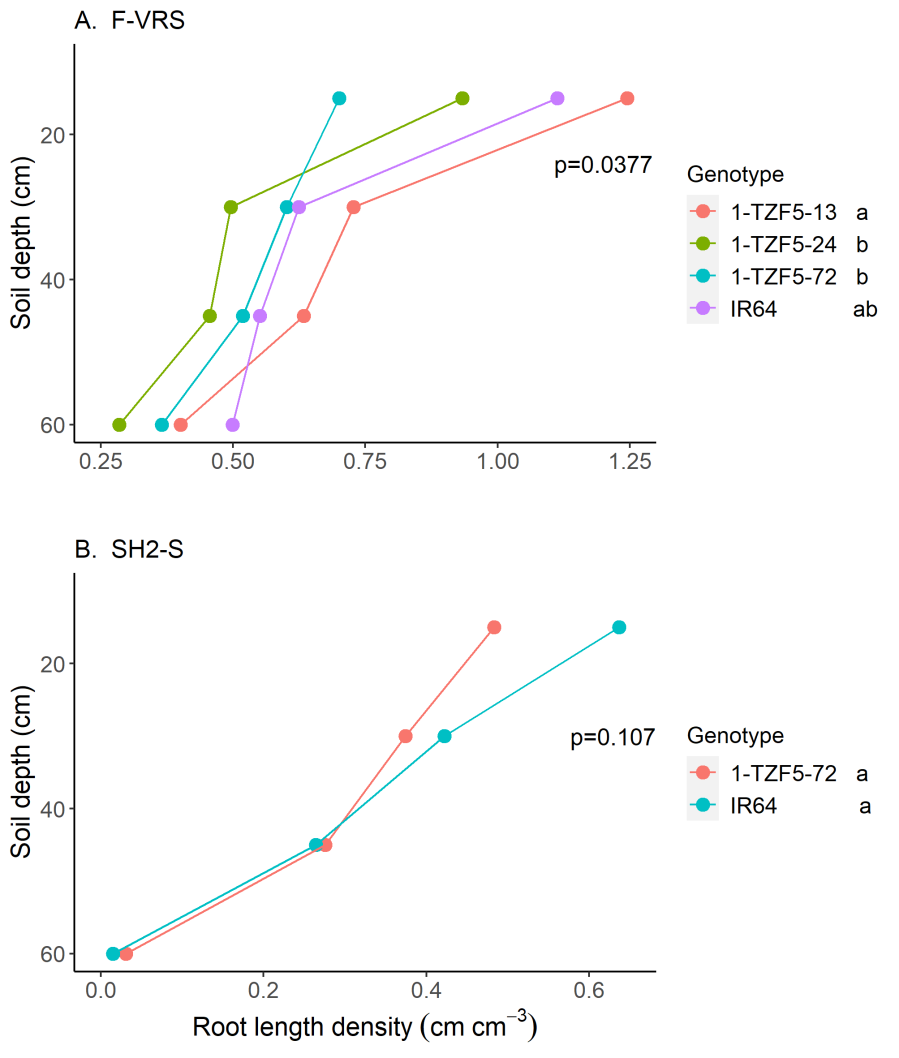


**Figure S16.** Transgenic lines - Root length density at four depth increments in IR64 and 1-TZF5-72 lines under drought stress in Trials F-VRS and SH2-S. (A, B) Root samples were taken at 101 das in Trial F-VRS and 118 das in SH2-S, at 3 locations per plot using a 4-cm-diameter core sampler (fabricated at IRRI, Los Baños, Philippines) to a depth of 60 cm. Soil cores were divided into 15-cm segments, and roots were washed by repeatedly mixing the soil with water in a container, and pouring the root-water suspension over a 1-mm plastic sieve. All samples were stored in 50% ethanol until scanning. Root samples were scanned at 600 dpi (Epson V700, California, USA), and scanned images were analyzed using WinRhizo v. 2007d (Régent Instruments, Québec, Canada). Root length density was calculated as the total root length per 15-cm soil core segment divided by the volume of the soil core segment (188.5 cm^3^). Mean values of 4 replicates are presented and were compared by ANOVA across depths. Significant differences among lines are indicated by the letters next to the legend in each panel.

SH: Screenhouse, F: Field

IR64: drought susceptible wild-type,  1-TZF5-13, 1-TZF5-24, 1- T-TZF5-72: transgenic lines

W: well-watered, R: reproductive stage drought stress, VRS: vegetative and reproductive stage drought stress

**
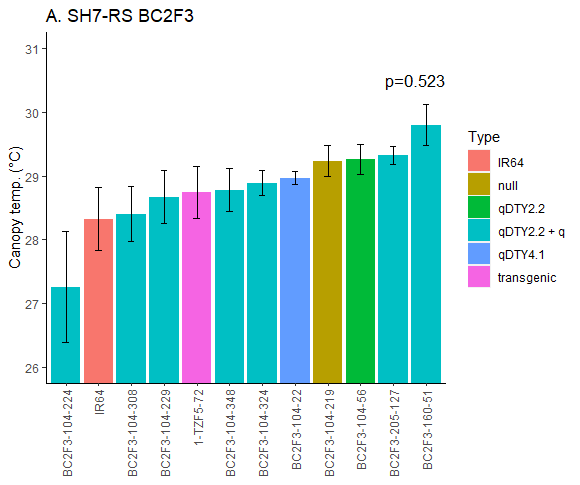
**

**
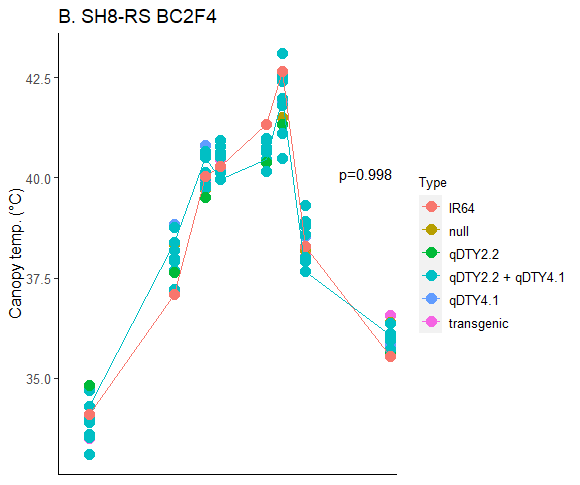
**

**
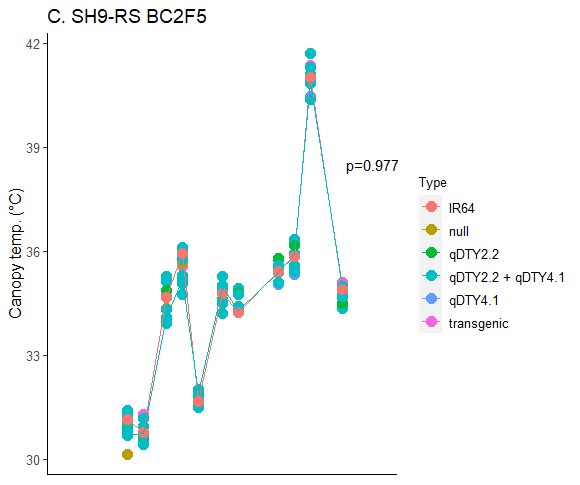
**

**Figure S17.** Pyramided lines: canopy temperature across each drought trial (A. Trial SH7, B. Trial SH8, and C. Trial SH9). Symbols represent means per line or genotype group. No significant differences among genotype groups were observed across measurement dates.

SH: screenhouse

W: well-watered, RS: reproductive stage drought stress

IR64: drought susceptible wild-type, 1- T-TZF5-72: transgenic line, qDTY2.2: pyramided line with qDTY2.2 and OsTZF5, qDTY2.2 + qDTY4.1: pyramided line with qDTY2.2, qDTY 4.1 and OsTZF5, qDTY4.1: pyramided line with qDTY4.1 and OsTZF5

**
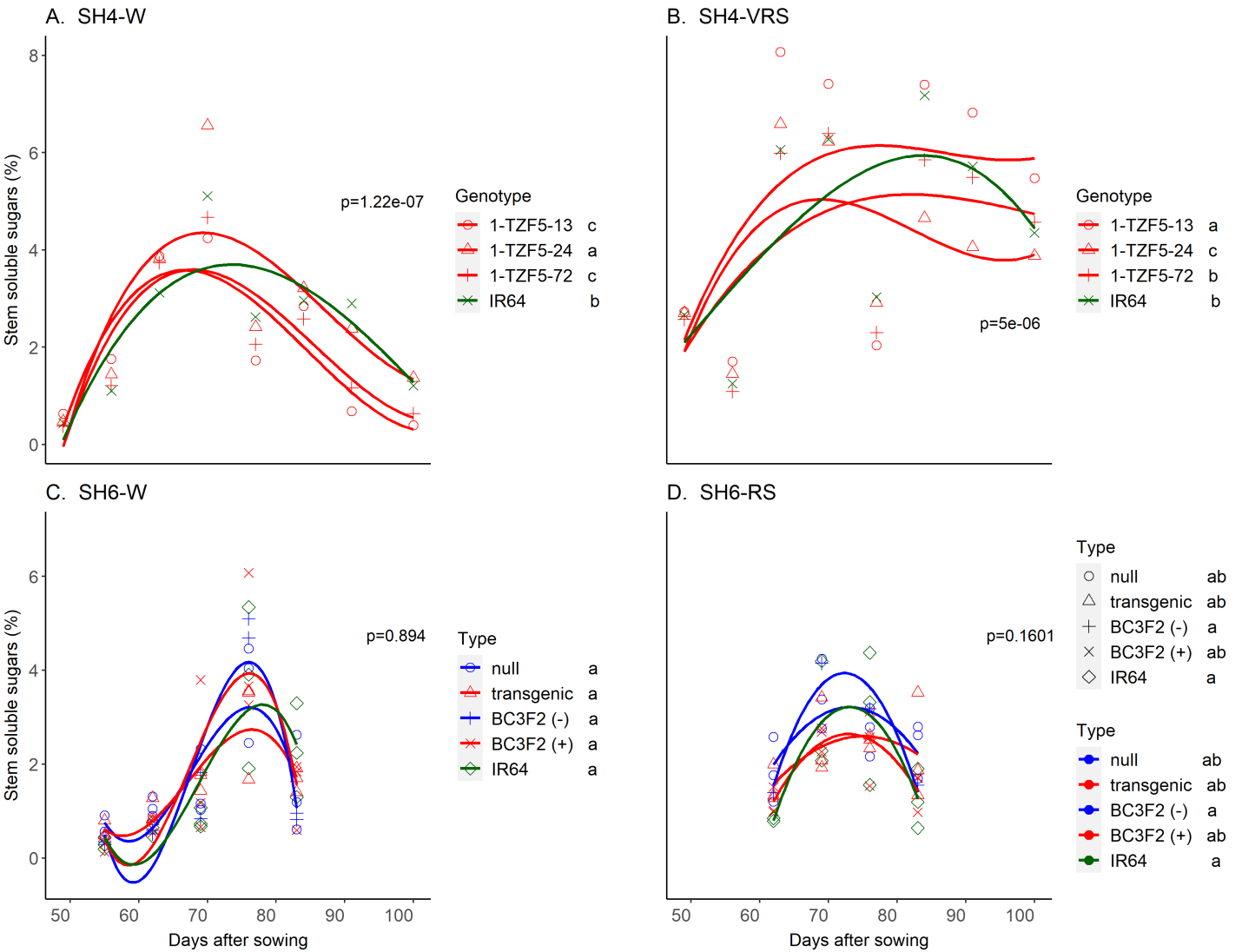
**

**Figure S18**. Stem soluble sugar concentrations at vegetative and reproductive stages in screenhouse trials SH4 and SH6. Measurements were performed on plants grown under well-watered (A) and drought stress conditions (B). Symbols represent means (*n* = 4) per transgenic line or genotype group and were compared across dates by ANOVA. Significant differences among lines/genotype groups are indicated by the letters next to the legend in each panel.

SH: screenhouse

W: well-watered, RS: reproductive stage drought stress, VRS: vegetative and reproductive stage drought stress

IR64: drought susceptible wild-type, BC3F1(-): background-cleaned lines without the transgene, BC3F1(+): background-cleaned lines with the transgene, transgenic: 1-TZF5-13, 1-TZF5-24, and 1- T-TZF5-72

**
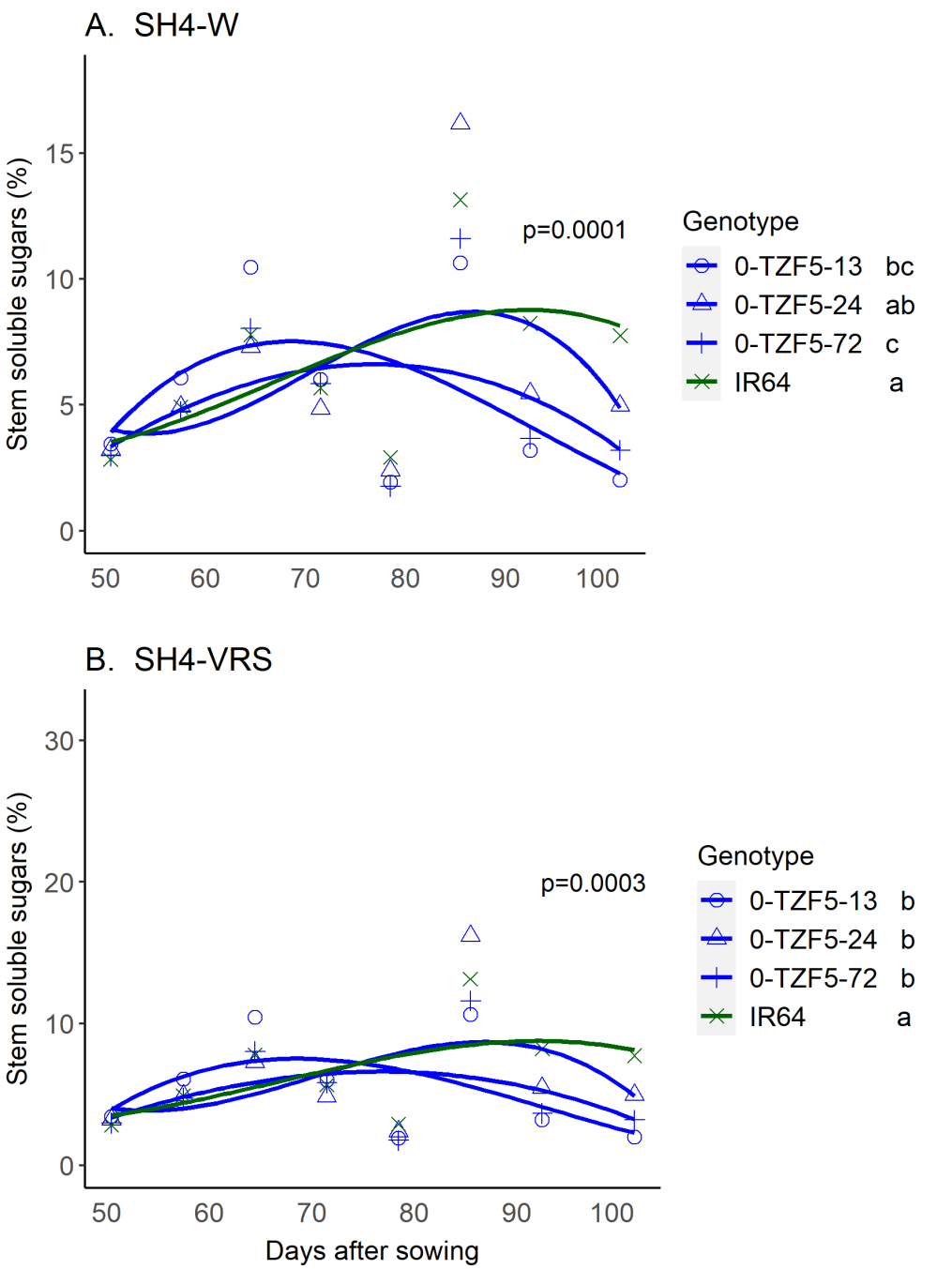
**

**Figure S19**. Null lines - Stem soluble sugar concentrations at vegetative and reproductive stages in screenhouse trial SH4. Measurements were performed on plants grown under well-watered (A) and drought stress conditions (B). Symbols represent means (*n* = 4) per null line and IR64 and were compared across dates by ANOVA. Significant differences among lines/genotype groups are indicated by the letters next to the legend in each panel.

SH: screenhouse

W: well-watered, RS: reproductive stage drought stress, VRS: vegetative and reproductive stage drought stress

IR64: drought susceptible wild-type, BC3F1(-): background-cleaned lines without the transgene, BC3F1(+): background-cleaned lines with the transgene, transgenic: 1-TZF5-13, 1-TZF5-24, and 1- T-TZF5-72
